# Guidelines development protocol and findings: part of the 2021 Australian evidence-based guidelines for diabetes-related foot disease

**DOI:** 10.1186/s13047-022-00533-8

**Published:** 2022-04-19

**Authors:** Peter A. Lazzarini, Anita Raspovic, Jenny Prentice, Robert J. Commons, Robert A. Fitridge, James Charles, Jane Cheney, Nytasha Purcell, Stephen M. Twigg, on behalf of the Australian Diabetes-related Foot Disease Guidelines & Pathways Project

**Affiliations:** 1 School of Public Health and Social Work, Queensland University of Technology, Brisbane, Australia; 2 Allied Health Research Collaborative, The Prince Charles Hospital, Brisbane, Australia; 3 School of Allied Health, Human Services and Sport College of Science, Health & Engineering, La Trobe University, Bundoora, Australia; 4 Hall and Prior Health and Aged Care Group, Perth, Australia; 5 Internal Medicine Services, Ballarat Health Services, Ballarat, Victoria Australia; 6 Global Health Division, Menzies School of Health Research, Charles Darwin University, Darwin, Northern Territory Australia; 7 Vascular and Endovascular Service, Royal Adelaide Hospital, Adelaide, Australia; 8 Discipline of Surgery, The University of Adelaide, Adelaide, Australia; 9 First Peoples Health Unit, Faculty of Health, Griffith University, Gold Coast, Australia; 10 Diabetes Victoria, Melbourne, Australia; 11 Diabetes Feet Australia, Brisbane, Australia; 12 Australian Diabetes Society, Sydney, Australia; 13 Sydney Medical School (Central), Faculty of Medicine and Health, University of Sydney, Sydney, Australia; 14 Department of Endocrinology, Royal Prince Alfred Hospital, Camperdown, Australia

**Keywords:** Classification, Diabetes-related foot disease, Diabetic foot, Guidelines, Infection, Offloading, Peripheral artery disease, Peripheral neuropathy, Ulcers, Wounds

## Abstract

**Background:**

Diabetes-related foot disease (DFD) is a leading cause of the Australian disease burden. The 2011 Australian DFD guidelines were outdated. We aimed to develop methodology for systematically adapting suitable international guidelines to the Australian context to become the new Australian evidence-based guidelines for DFD.

**Methods:**

We followed the Australian National Health Medical Research Council (NHMRC) guidelines for adapting guidelines. We systematically searched for all international DFD guideline records. All identified records were independently screened and assessed for eligibility. Those deemed eligible were further assessed and included if scoring at least moderate quality, suitability and currency using AGREE II and NHMRC instruments. The included international guidelines had all recommendations extracted into six sub-fields: prevention, wound classification, peripheral artery disease, infection, offloading and wound healing. Six national panels, each comprising 6–8 multidisciplinary national experts, screened all recommendations within their sub-field for acceptability and applicability in Australia using an ADAPTE form. Where panels were unsure of any acceptability and applicability items, full assessments were undertaken using a GRADE Evidence to Decision tool. Recommendations were adopted, adapted, or excluded, based on the agreement between the panel’s and international guideline’s judgements. Each panel drafted a guideline that included all their recommendations, rationale, justifications, and implementation considerations. All underwent public consultation, final revision, and approval by national peak bodies.

**Results:**

We screened 182 identified records, assessed 24 full text records, and after further quality, suitability, and currency assessment, one record was deemed a suitable international guideline, the International Working Group Diabetic Foot Guidelines (IWGDF guidelines). The six panels collectively assessed 100 IWGDF recommendations, with 71 being adopted, 27 adapted, and two excluded for the Australian context. We received 47 public consultation responses with > 80% (strongly) agreeing that the guidelines should be approved, and ten national peak bodies endorsed the final six guidelines. The six guidelines and this protocol can be found at: https://www.diabetesfeetaustralia.org/new-guidelines/

**Conclusion:**

New Australian evidence-based guidelines for DFD have been developed for the first time in a decade by adapting suitable international guidelines. The methodology developed for adaptation may be useful for other foot-related conditions. These new guidelines will now serve as the national multidisciplinary best practice standards of DFD care in Australia.

**Supplementary Information:**

The online version contains supplementary material available at 10.1186/s13047-022-00533-8.

## Background

Diabetes-related foot disease (DFD) is a leading cause of morbidity, mortality and healthcare cost burdens in Australia [1–4]. DFD is defined as foot ulceration, infection, or tissue destruction in people with diabetes, accompanied by the risk factors of peripheral neuropathy (PN) and/or peripheral artery disease (PAD) [4–6]. Each year DFD affects approximately 50,000 Australians, with a further 300,000 having risk factors for developing DFD [1–4]. Although DFD causes a large disease burden, Australian regions that have systematically introduced multi-disciplinary foot care services which adhered to evidence-based DFD guideline recommendations, have significantly reduced their burden of DFD [7–9].

A key recommendation of the *Australian DFD Strategy 2018–2022* was to ensure Australia has national DFD guidelines that continually reflect up-to-date robust evidence to guide multi-disciplinary standards of DFD care [1, 2]. However, Australia’s most recent 2011 national evidence-based DFD guideline [10] is out-dated by world standards [11] and has been rescinded by the National Health and Medical Research Council (NHMRC) [12]. Thus, there was an urgent need to develop contemporary national guidelines for DFD [1, 2].

The NHMRC Guidelines for Guidelines recommends developing new guidelines either from scratch (‘de novo’), or adopting and/or adapting other suitable high-quality international guidelines if no Australian equivalent is available [13]. With no known Australian DFD guidelines under development [14], and a low likelihood of acquiring the estimated AU$1 million needed to develop a guideline de novo [15], Diabetes Feet Australia (DFA) appointed a multi-disciplinary Guideline development working group to oversee a project to adopt or adapt suitable international guidelines. Members of the group (“the authors”) were invited based on having an (inter)nationally-recognised DFD guideline and/or research publication track record, or being a consumer or Aboriginal and Torres Strait Islander representative with expertise in DFD [16, 17]. The aim was to identify and adapt suitable international source guidelines to the Australian health context to become the new multi-disciplinary Australian evidence-based guidelines for DFD for all Australian health professionals.

## Methods

The methodology for developing this guideline followed eight overarching methodological steps that aligned with best practice principles for adapting suitable international source guidelines as recommended by the NHMRC Guidelines for Guidelines [13] and the ADAPTE and GRADE-ADOLOPMENT frameworks [17, 18]. The eight overarching steps and our approach to implementing these steps are detailed below. In summary the steps were: i) defining the scope of the guidelines; ii) identifying potential international source guidelines; iii) determining suitable international source guidelines to adapt; iv) deciding which recommendations to adopt, adapt, or exclude; v) drafting recommendations and the reasoning for those recommendations; vi) developing guideline manuscripts; vii) external consultation and approval of guideline manuscripts; and viii) developing clinical pathways to aid implementation into practice.

### Defining the scope of the guidelines

The scope of these guidelines were defined using the PIPOH (spelt out below) framework recommended by ADAPTE [17] and based on (inter)national DFD reporting standards [1, 5, 6, 19], i.e.:

- Population(s) of interest - were those defined as at risk of, or with, DFD [5, 6];
- Intervention(s) of interest - were those interventions typically used to screen, diagnose, prevent, or treat the population(s) of interest [5, 6];
- Professions to be targeted - were those multiple medical, surgical, nursing and allied health disciplines that typically provide prevention or treatment for the population(s) of interest [1, 19, 20];
- Outcomes of interest - were those outcome measures typically used for the population(s) of interest, such as ulcer healing or amputation [5, 6];
- Health care context to be targeted - were those secondary and/or tertiary health care settings and organisations that typically provide prevention or treatment for the population(s) of interest in Australia [1, 19, 20].

### Identifying potential international source guidelines

Based on the above defined scope we performed a systematic search for potentially suitable international source guidelines [13, 17]. The search strategy included any guideline record published until 1 May 2020, in the International Guidelines Library [21] or Australian Clinical Practice Guidelines Register databases [14]. These guideline databases were chosen as they were specifically recommended for this purpose by either NHMRC [13] or ADAPTE [17]. We used the following free text search terms in these databases: “diabetes”, “foot”, “feet”, “wound” or “ulcer”. All authors were also asked to identify any other potentially suitable guideline records of which they were aware, and these were included in the search strategy as additional records identified via other sources.

The title and abstract (if available) of each unique record identified from the search strategy was independently screened by three authors (PAL, AR, JP) for eligibility for full-text assessment. The inclusion criteria were those records: i) with the primary aim of developing clinical guidelines to prevent or manage people with, or at risk of, DFD; ii) developed for an international multi-disciplinary health professional audience; iii) written in English (the national language of Australia); iv) based on systematic review(s) of the available literature; and v) which incorporated a final systematic review search date within 3 years of our search date (i.e. 1 May 2017) for currency. All records screened as eligible by any of the three authors were included for full text assessment.

Each guideline record identified as eligible after screening then had their full text retrieved and assessed based on the same above inclusion criteria by two authors independently (PAL, AR, or JP) [22]. Any disagreements on eligibility by the two authors were discussed until consensus was reached or if unable to be reached a third author decided [22]. Decisions to exclude any full-text records were recorded identifying the criteria the guideline record failed to meet [17, 22].

### Determining suitable international source guidelines to adapt

Remaining eligible full text records were then independently assessed by four authors (PAL, AR, JP, RJC) for their methodological quality, suitability and currency to be adopted or adapted to the Australian health context [13, 17]. The Appraisal of Guidelines for Research and Evaluation II (AGREE II) Instrument was used to assess methodological quality [17, 23, 24]. The AGREE II instrument is a widely used, valid and reliable 23-item instrument, each using a 7-point Likert scale, for assessing methodological quality of guidelines [23, 24]. The scores of the four authors were summed and divided by the maximum possible score to determine a total score % [24]. Scores were categorised: high quality if scored > 70%, moderate if 50–69%, and low if < 50% [17, 23, 24].

A customised tool from NHMRC was used to assess suitability and currency [13]. The tool is a 22-item tool developed by the authors using the exact questions outlined in the NHMRC Guidelines for Guidelines table of factors that should be considered for assessing the suitability and currency of a guideline to adopt or adapt in the Australian context [13] (Supplementary Material Table S1). The tool included 21 items using a 7-point Likert scale to determine suitability, and, one open item using the final literature search date of the guideline record to determine currency. Total scores for suitability were determined using the same formula used for the AGREE II tool [23, 24] and categorised: high suitability if scored > 70%, moderate if 50–69%, and low if < 50%. Currency was defined as the total time elapsed between the final literature search date of the guideline record and the search strategy date of this protocol (i.e. 1 May 2020), and categorised: high currency if < 1 year since final search date, moderate currency if < 3 years, and low currency if > 3 years [13, 17].

All documents that informed the development of each eligible full text guideline record were included as part of these assessments, including any systematic reviews, methodology protocols and technical reports [13, 17]. Any record deemed as having at least moderate quality, moderate suitability and moderate currency following these assessments was defined and included as a suitable international source guideline to adopt or adapt to the Australian health context for this project [13, 17].

### Deciding which recommendations to adopt, adapt or exclude

All recommendations within the above suitable international source guidelines were individually extracted and evaluated to determine if they should be adopted, adapted, or excluded in the Australian context [13, 17, 18]. The following five sub-steps were followed: a) recommendations were categorised into six sub-fields; b) national expert panels were convened for each sub-field; c) panels screened all recommendations in their sub-field; d) panels assessed any recommendations if unsure of acceptability or applicability; and e) panels decided which recommendations to adopt, adapt or exclude [13, 17, 18].

- Recommendations were categorised into six sub-fields

Two authors (PAL, AR) independently categorised all recommendations (and the relevant clinical questions they addressed) from all included suitable source guidelines into one of six DFD sub-fields that the authors considered the recommendation was primarily addressing [5, 6]. The six sub-fields aligned with international DFD standards and included: prevention, wound classification, peripheral artery disease (PAD), infection, offloading, and wound healing interventions [5, 6]. The two authors discussed any disagreements until consensus was reached or if this was unable to be reached then a third author decided [22].

b. National expert panels were convened for each sub-field

All recommendation(s) (and all relevant documentation of reasoning informing the recommendation(s), including rationale, summary(s) of evidence, evidence statements, quality of evidence summaries, risk of bias tables, evidence tables), were forwarded to the relevant sub-field national expert panel to screen. Each national expert panel comprised 6–8 members and was chaired by an author with relevant (inter)national research and/or clinical practice expertise in the sub-field. Panel membership included 4–6 members with either (inter)national research and/or clinical practice sub-field expertise from different disciplines, states (or territories) and genders, plus, a consumer and an Aboriginal and Torres Strait Islander representative with expertise in DFD. An (inter)national research expert was defined as having published in peer-reviewed journals and/or been ranked as an Australian expert in the sub-field according to Expertscape [25]. An (inter)national clinical practice expert was defined as having presented at an (inter)national conference and/or been a member of an (inter)national committee in the sub-field.

c. Panels screened all recommendations in their sub-field

At least two panel members independently screened each recommendation (and all relevant documentation informing the recommendation) in their sub-field for acceptability and applicability in the Australian health context, using a customised ADAPTE evaluation form (Supplementary Material Fig. S1) [17]. The ADAPTE form comprised 7-items, each using a 3-point Likert scale (yes, unsure, or no), organised into the domains of acceptability and applicability [17]. Acceptability items included screening the quality of evidence, strength of recommendation and user (patients and providers in the context) values ratings for the recommendation to determine if the panel agreed with the included guideline’s original ratings [17]. Applicability items included screening the applicability to patients, availability of equipment and expertise, and any legislative or policy constraints for the recommendation in the Australian health context [17]. The Australian health context was defined as individual patients with, or at risk of, DFD, attending the multiple health professional disciplines that typically provide prevention or treatment services in the secondary and tertiary Australian health care settings that house those services [1, 19, 20]. Any disagreements on item scores were discussed by the two members until consensus was reached, or if it could not be reached, a third member decided [22]. All panel members then met to discuss and decide by consensus all ratings for each item in each recommendation. All recommendations that scored “yes” agreement in all items were able to be adopted for the Australian context. Any recommendations scoring any items as “unsure” or “no” agreement in one or more items required full assessment.

d. Panels assessed any recommendations if unsure of acceptability or applicability

All recommendations requiring full assessment were done so using a customised Grading of Recommendations Assessment, Development and Evaluation (GRADE) Evidence to Decision (EtD) template (Supplementary Material Fig. S2) [18, 26, 27]. This involved one panel member systematically extracting and populating the template verbatim with all relevant text relating to the rationale for that recommendation from the source guideline for eight important EtD criteria: the problem, desirable effects, undesirable effects, quality of evidence, values, balance of effects, acceptability and feasibility [18, 26, 27]. The same member also added any additional relevant Australian considerations to each of the eight criteria that they considered necessary to inform the Australian context from relevant literature and/or their expert opinion. The populated EtD was checked for accuracy by another member and any disagreements discussed between the two members until agreement was reached. Based on the populated EtD, one panel member rated the detailed and summary judgement items in each of the eight important EtD criteria [18, 26, 27]. Another member checked all judgements made by the first member with any disagreements discussed until consensus was reached [22]. All panel members then met to discuss and decide by consensus all summary judgement item ratings for each of the eight important EtD criteria for each recommendation. Finally, the panel compared the level of agreement between their eight EtD summary judgement items with the source guideline’s summary judgement items (if able to be determined) to determine the level of agreement for each item as: yes (agreed), unsure, or no (disagreed) [18].

e. Panels decided which recommendations to adopt, adapt or exclude

The decision to adopt, adapt, or exclude each recommendation was made based on the level of agreement between the panel’s consensus summary judgement items and that of the source guideline’s summary judgement items for each EtD item in each recommendation [18]. This was performed via discussion and a consensus decision by all panel members after reviewing their completed ADAPTE form and/or GRADE EtD summary judgement items for each recommendation [17, 18]. A decision to adopt was made if all items in the customised ADAPTE form scored “yes (agreed)” and/or the panel’s GRADE EtD summary judgement items generally agreed with the source guideline’s summary judgement items [18]. A decision to adapt was made if the ADAPTE form scored an “unsure” or “no” on any item, and there was disagreement between the panel’s GRADE EtD summary judgement items and that of the source guideline’s summary judgement items [18]. A decision to exclude was made if the ADAPTE form scored an “unsure” or “no” on any item, there were substantial disagreements between the panel’s GRADE EtD summary judgement items and that of source guideline’s, and/or the panel considered the recommendation was not acceptable or applicable to use in the Australian context.

### Drafting recommendations and reasoning for those recommendations

All recommendations and supporting reasoning for the recommendations were then drafted via consensus by each panel. Depending on the panel’s decision to adopt, adapt or exclude the source guideline’s recommendation shaped how the panel drafted each recommendation [18]. When adopting a recommendation, the panel re-stated the source guideline’s recommendation verbatim (including the quality of evidence and strength of recommendation) [18]. Minor wording changes were only permitted if the panel felt exchanging an Australian term for an international term was necessary to improve the interpretation of the recommendation without changing the concept. When adapting a recommendation, the panel drafted the recommendation based on the source guideline’s recommendation and adapted/edited the recommendation’s wording to reflect the panel’s specific difference in judgement(s) to that of the source guideline’s judgements [18]. As recommended by GRADE the panel drafted each adapted recommendation with the aim of being clear, specific and unambiguous on what is recommended, for which persons, and under what circumstances [18, 26–30]. Further, the panel by consensus, re-evaluated the quality of evidence using the GRADE system as High, Moderate, Low, or Very Low, based on the panel’s level of confidence that the findings were from studies that reported consistent effects with low risk of bias and further research was unlikely to change that confidence [26, 27]. The panel also rated the strength of recommendation using the GRADE system, based on weighing up the balance of effects, quality of evidence, applicability and feasibility [26, 27] in the Australian health context as: Strong, if there was a large clear difference in the balance of effects (i.e. large net benefit or net harm) between an intervention and control; or Weak, if there was a small and/or uncertain difference [26, 27]. When excluding a recommendation, the panel simply stated the recommendation was excluded.

Regardless of the panel’s decision to adopt, adapt, or exclude the recommendation, the panel drafted transparent reason sections to support their decisions for each recommendation [18]. These sections included: rationale for the decision, justifications for the recommendation, and considerations on implementation, special subgroups, monitoring and future research priorities for the recommendation [18, 26–30]. The rationale for the decision involved the panel documenting why they decided to adopt, adapt, or exclude the recommendation based on the similarities or differences in judgements with those of the source guideline’s judgements. The panel also clearly outlined any wording changes as compared to the source guideline’s original recommendation [18]. The justifications for the recommendation were based on the panel carefully weighing up the panel’s ADAPTE and/or GRADE EtD summary judgements to determine the strength of the recommendation, quality of evidence rating, patient (and provider) values and preferences, and acceptability and feasibility for the Australian health context [18, 26–30]. Additionally, for those recommendations that were adapted or excluded, where applicable the panel also outlined their detailed judgements for each of the eight important GRADE EtD criteria: the problem, values, desirable effects, undesirable effects, balance of effects, quality of evidence, acceptability, and feasibility [18, 26, 27]. Lastly, based on the source guideline’s considerations, literature reviews and expert opinion, the panel outlined any important considerations for health professionals to consider when implementing the recommendations [18, 26–30]. These considerations included: implementing the recommendation in the Australian health context, implementing in special subgroups (including in geographically remote, Aboriginal and Torres Strait Islander, and potentially contraindicated subgroups), monitoring the implementation, and any future research priorities for the recommendation [18, 26–30].

### Developing guideline manuscripts

Each panel’s sub-field guideline manuscript was developed using an introduction, methods, results, and discussion framework. The introduction and methods were brief summaries of the introduction and methods contained in this guideline development protocol manuscript, along with any other relevant sub-field literature. The results sections were a collation of all the panel’s consensus recommendations and supporting reasoning (as described in Section v). The discussion sections typically included summaries of the similarities and differences between the new Australian guideline, previous Australian guideline, and the source guideline in terms of recommendations and rationale. The final draft guideline manuscript was approved via consensus of each panel.

Each draft guideline manuscript was then peer-reviewed by at least one author not involved in the sub-field panel, to identify any obvious discrepancies in the recommendations or supporting reasons for the recommendations. If the author(s) identified any discrepancies, the panel was asked to revise the manuscript to consider and address the discrepancy. The agreed final draft of the sub-field guideline manuscript was deemed the consultation draft.

### External consultation and approval of guideline manuscripts

The six draft guideline manuscripts used for public consultation (also known as “chapters”), plus this guideline development protocol manuscript, were collated and formatted for consistency ready for public consultation as the new Australian evidence-based guidelines for DFD.. Additionally, a customised public consultation survey based on example surveys from the ADAPTE framework were developed for each guideline to more efficiently gain and collate aggregated feedback from the public consultation process [17] (Supplementary Material Table S2). Finally, the ADAPTE Checklist for Adapted Guideline Content was completed by the authors to ensure all guideline elements had been completed [17].

Public consultation was targeted towards Australian health professionals assessing and managing patients with DFD as well as national peak bodies/organisations representing health professionals, consumers or Aboriginal and Torres Strait Islanders. The consultation material included the six guideline manuscripts, the guideline development protocol manuscript and the consultation surveys. Public consultation for each guideline was open for a minimum period of 4 weeks and notification of the consultation period was posted weekly on DFA and/or Australian Diabetes Society (ADS) web and social media sites, plus, invitations were sent electronically to relevant national peak bodies for health professionals, consumers or Aboriginal and Torres Strait Islander people. Any Australian health professional or national peak body with an interest was encouraged to respond.

At the conclusion of the public consultation period all consultation survey responses were collated and analysed. The authors disseminated all aggregated survey findings and feedback comments to the relevant sub-field guideline panels for consideration and revision of their respective manuscripts accordingly. The authors subsequently quality checked each panel’s revisions [17]. Any substantial disagreements with the panel’s revisions were discussed between the authors and the panel concerned until consensus was reached. All aggregated consultation survey findings and each panel’s response to feedback received were publicly displayed on the DFA website. Endorsement of the guidelines were finally specifically sought from DFA, ADS, and Diabetes Australia, plus, invitations to endorse were sent to all aforementioned relevant national peak bodies. All final endorsed guideline manuscripts were publicly published online in full on the DFA website https://www.diabetesfeetaustralia.org/new-guidelines/ [31–36], submitted to peer-reviewed journals for publication and registered on the International Guidelines Library [21] and Australian Clinical Practice Guidelines register [14].

These new Australian guidelines will be reviewed every 2 years by the authors, or equivalent national guidelines committee, to determine by consensus if any substantial new evidence has been published that contradicts or substantially enhances any existing recommendations. If that is deemed to be the case, or after 4 years (i.e. 2025), whichever comes first, we recommend updating these guidelines using similar adaptation methodology used to develop these new guidelines or develop new guidelines de novo if the requisite funding becomes available.

### Developing clinical pathways to aid implementation into practice

To try and facilitate improved implementation into clinical practice, clinical pathways were developed for each guideline that incorporated the recommendations from that guideline. The process used for developing the clinical pathways was that advocated by Flores et al. (2019) [37]. This process included: each panel extracted any clinical pathways from their source guidelines as examples; the guidelines group developed a clinical pathway template based on these example pathways and similar national diabetes clinical pathways; each panel then used the template to develop their clinical pathway(s) by populating the template with their recommendations; and finally, the guidelines group reviewed all the clinical pathways for final quality assurance, formatting and consistency checks [37].

## Results

### Identifying potential international source guidelines

**Fig. 1:**
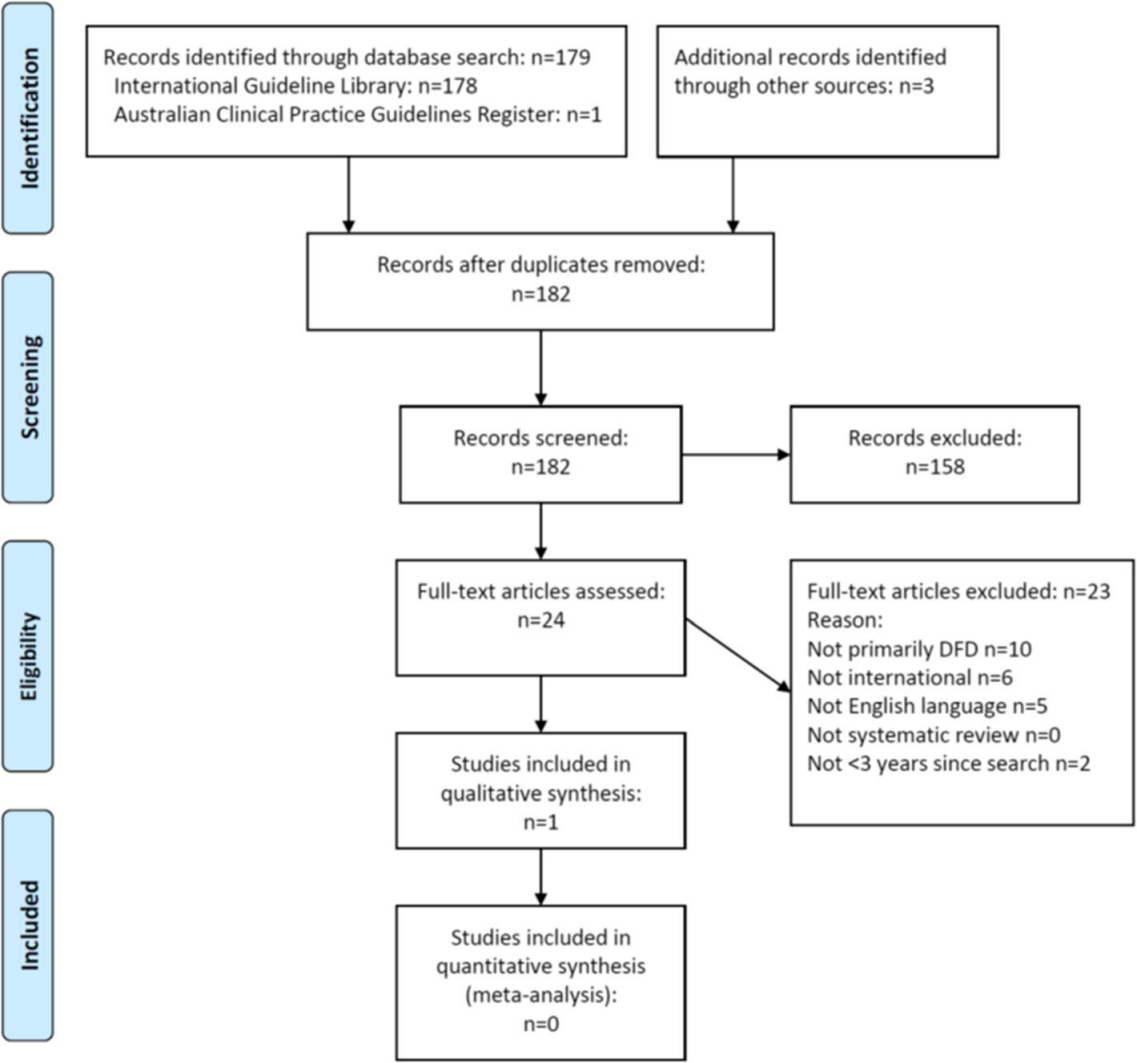
Preferred reporting items for systematic reviews and meta-analyses flow diagram

Figure 1 displays the flowchart of results from the search strategy, which yielded a total of 182 relevant unique guideline records. After title and abstract screening, 158 records were excluded with 24 records remaining for full text assessment. After full text assessment, the only guideline deemed eligible to be assessed to determine if it was a suitable international source guideline to adapt to the Australian health context was the 2019 International Working Group on the Diabetic Foot (IWGDF) Guidelines [38–44].

### Determining suitable international source guidelines to adapt

Table 1 displays the quality assessments of the IWGDF guidelines. The total quality score category for the IWGDF guidelines was rated as having high methodological quality. All 23 items also scored high quality ratings, except for four applicability items and one rigour of development item that scored moderate quality, and one stakeholder involvement item that scored low quality. Table 2 displays the suitability and currency assessments of the IWGDF guideline. The total suitability score category for the IWGDF guidelines was rated as having high suitability to the Australian health context. All 21 suitability items also scored high suitability ratings, except two implementability items that scored moderate suitability. Finally, the currency score category for the only currency item for the IWGDF guidelines was rated as having moderate currency. Thus, with high quality, high suitability and moderate currency the 2019 IWGDF guidelines were determined to be a suitable international source DFD guideline to adapt to the Australian health context.

**Table 1:** Quality assessments of IWGDF guideline to adopt or adapt; using a customised AGREE II instrument*

Item No.	Item description	Assessor 1	Assessor 2	Assessor 3	Assessor 4	Total score	Total score %	Quality category^
Scope and purpose								
1	The overall objective(s) of the guideline is (are) specifically described	6	7	6	6	25	89%	High
2	The health question(s) covered by the guideline is (are) specifically described	6	6	7	7	26	93%	High
3	The population (patients, public, etc.) to whom the guideline is meant to apply is specifically described.	6	6	7	7	26	93%	High
	**Domain Score** (sum of 3 items)	18	19	20	20	77	92%	High
Stakeholder involvement								
4	The guideline development group includes individuals from all relevant professional groups.	5	6	5	4	20	71%	High
5	The views and preferences of the target population (patients, public, etc.) have been sought.	3	2	1	2	8	29%	Low
6	The target users of the guideline are clearly defined.	5	5	7	6	23	82%	High
	**Domain Score** (sum of 3 applicable items)	13	13	13	12	51	61%	Moderate
Rigour of development								
7	Systematic methods were used to search for evidence.	7	7	7	7	28	100%	High
8	The criteria for selecting the evidence are clearly described.	7	7	7	7	28	100%	High
9	The strengths and limitations of the body of evidence are clearly described.	6	7	7	6	26	93%	High
10	The methods for formulating the recommendations are clearly described.	6	6	6	6	24	86%	High
11	The health benefits, side effects, and risks have been considered in formulating the recommendations.	5	7	7	6	25	89%	High
12	There is an explicit link between the recommendations and the supporting evidence.	5	6	7	6	24	86%	High
13	The guideline has been externally reviewed by experts prior to its publication.	5	5	4	5	19	68%	Moderate
14	A procedure for updating the guideline is provided.	5	6	5	6	22	79%	High
	**Domain Score** (sum of 8 items)	46	51	50	49	196	88%	High
Clarity of presentation								
15	The recommendations are specific and unambiguous.	6	6	7	7	26	93%	High
16	The different options for management of the condition or health issue are clearly presented.	6	6	7	6	25	89%	High
17	Key recommendations are easily identifiable.	7	7	7	7	28	100%	High
	**Domain Score** (sum of 3 items)	19	19	21	20	79	94%	High
Applicability								
18	The guideline describes facilitators and barriers to its application.	5	5	4	5	19	68%	Moderate
19	The guideline provides advice and/or tools on how the recommendations can be put into practice.	5	7	3	4	19	68%	Moderate
20	The potential resource implications of applying the recommendations have been considered.	5	5	2	4	16	57%	Moderate
21	The guideline presents monitoring and/or auditing criteria.	4	6	1	5	16	57%	Moderate
	**Domain Score** (sum of 4 items)	19	23	10	18	70	63%	Moderate
Editorial independence								
22	The views of the funding body have not influenced the content of the guideline.	7	7	7	7	28	100%	High
23	Competing interests of guideline development group members have been recorded and addressed.	6	7	7	6	26	93%	High
	**Domain Score** (sum of 2 items)	13	14	14	13	54	96%	High
Overall guideline assessment								
	Rate the overall quality of this guideline	**6**	**6**	**6**	**6**	**24**	**86%**	**High**
	I would recommend this guideline for use.	**Yes**	**Yes,with modifications**	**Yes**	**Yes**			
	**Total Guideline Score** (sum of all 23 individual items)	**128**	**139**	**128**	**132**	**527**	**82%**	**High**
	**Total Guideline Score %**	**80%**	**86%**	**80%**	**82%**			
	**Total Guideline Quality Category**	**High**	**High**	**High**	**High**			

*Each item is scored using a 7-point Likert-scale: 1 = lowest possible score, 7 = highest possible score

^Quality category definitions: High > 70%, Moderate 50–69%, and Low quality < 50% for total score %

**Table 2:** Suitability and currency assessments of IWGDF guideline to adopt or adapt; using a customised NHMRC table of factors*

Item No.	Item question	Assessor 1	Assessor 2	Assessor 3	Assessor 4	Total score	Total score %	Suitability category^
Relevance								
1	Is the clinical or public health context similar to Australia?	6	5	5	7	23	82%	High
2	Are the population, intended users and settings comparable?	6	6	7	7	26	93%	High
3	Are the recommended interventions available in Australia?	6	6	7	6	25	89%	High
4	Are the guideline questions relevant in the new (Australian) context?	6	7	7	7	27	96%	High
5	Do the values and preferences considered in the guideline reflect the new (Australian) context?	6	6	7	7	26	93%	High
6	Are relevant outcomes used?	6	7	7	7	27	96%	High
	**Domain Score** (sum of 6 items)	36	37	40	41	154	92%	High
Currency								
7	When was the evidence review conducted (i.e. final literature search date)?	July 2018	Oct 2018	July 2018	July 2018	< 3 years	Moderate	Moderate (Currency)#
8	Is the evidence contained out of date?	6	7	6	6	27	96%	High
9	Are new studies’ findings conducted since the review likely to change the evidence?	6	7	6	6	27	96%	High
10	Has new evidence superseded the information contained in the recommendations?	6	7	6	6	27	96%	High
11	Does new evidence contradict the recommendations?	6	7	6	6	27	96%	High
	**Domain Score** (sum of 4 applicable items)	24	28	24	24	108	96%	High
Trustworthiness								
12	Is there a detailed description of the development process?	7	7	7	7	28	100%	High
13	Were conflicts of interest declared and managed?	6	7	7	6	26	93%	High
14	Was a grading system used for the recommendations?	6	7	7	7	27	96%	High
15	Are the evidence tables clearly laid out and accurate?	6	7	7	6	26	93%	High
16	Was the evidence review systematic and well-documented?	7	7	7	7	28	100%	High
	**Domain Score** (sum of 5 items)	32	35	35	33	135	96%	High
Access to evidence								
17	Are the tables detailing the source evidence (e.g. GRADE Evidence to Decision tables) available?	6	7	7	7	27	96%	High
18	Can permission be sought to use these tables?	6	7	7	7	27	96%	High
	**Domain Score** (sum of 2 items)	12	14	14	14	54	96%	High
Implementability								
19	Is information provided in the guideline to assist implementation?	4	6	3	5	18	64%	Moderate
20	Are steps taken to improve the guideline’s implementability?	4	6	2	5	17	61%	Moderate
	**Domain Score** (sum of 2 items)	8	12	5	10	35	63%	Moderate
Acceptability								
21	Are the recommendations acceptable?	6	7	7	7	27	96%	High
22	Do the recommendations relate to current practice?	6	6	7	7	26	93%	High
	**Domain Score** (sum of 2 items)	12	13	14	14	53	95%	High
	**Total Guideline Score** (sum of all 21 applicable items)	**124**	**139**	**132**	**136**	**531**	**90%**	**High**
	**Total Guideline Score %**	**84%**	**95%**	**90%**	**93%**			
	**Total Guideline Suitability Category**	**High**	**High**	**High**	**High**			

*Each item is scored using a 7-point Likert-scale: 1 = lowest possible score, 7 = highest possible score

^Suitability category definitions: High > 70%, Moderate 50–69%, and Low suitability < 50% for total score %

#Currency category definitions: High < 1 year, Moderate 1–3 years, and Low currency > 3 years since systematic review search date

### Deciding which recommendations to adopt, adapt or exclude

Table 3 lists all final members of the six national expert panels, alongside the consumer and Aboriginal and Torres Strait Islander expert representatives. A total of 30 (inter)national DFD expert members from all Australian states/territories (except Australian Capital Territory) representing seven health profession disciplines were on these panels, including 12 podiatrists, five vascular surgeons, four wound nurses, three endocrinologists, three infectious diseases physicians, two orthopaedic surgeons and one pedorthist.

**Table 3:** National DFD expert panel members (discipline, state) for each sub-field panel

Criteria^a^	Prevention	Classification	PAD	Infection	Offloading	Wound Healing
Expert (Chair)	**Dr Anita Raspovic** (Podiatrist, VIC)	**Prof Stephen Twigg** (Endocrinologist, NSW)	**Prof Robert Fitridge** (Vascular Surgeon, SA)	**Dr Robert Commons** (ID Physician, VIC)	**A/Prof Peter Lazzarini** (Podiatrist, QLD)	**Dr Jenny Prentice** (Wound Care Nurse, WA)
Expert (Secretary)	**Dr Michele Kaminski** (Podiatrist, VIC)	**Dr Emma Hamilton** (Endocrinologist, WA)	**Prof Vivienne Chuter** (Podiatrist, NSW)	**Dr Robert Commons** (ID Physician, VIC)	**Dr Malindu Fernando** (Podiatrist, QLD)	**Ms Pam Chen** (Podiatrist, TAS)
Expert (Member)	**Prof Jonathan Golledge** (Vascular Surgeon, QLD)	**Dr Byron Perrin** (Podiatrist, VIC)	**Dr Frank Quigley** (Vascular Surgeon, QLD)	**Dr Sarah Lynar** (ID Physician, NT)	**Dr Mark Horsley** (Orthopaedic Surgeon, NSW)	**Prof Keryln Carville** (Wound Care Nurse, WA)
Expert (Member)	**Dr Joel Lasschuit** (Endocrinologist, NSW)	**Ms Hayley Ryan** (Wound Care Nurse, NSW)	**Dr Carsten Ritter** (Vascular Surgeon, WA)	**Dr Matthew Malone** (Podiatrist, NSW)	**Dr Brian Martin** (Orthopaedic Surgeon, NSW)	**A/Prof Peter Lazzarini** (Podiatrist, QLD)
Expert (Member)	**A/Prof Karl-Heinz Schott** (Pedorthist, NSW)	**Ms Jo Scheepers** (Podiatrist, WA)	**Dr Patrik Tosenovski** (Vascular Surgeon, WA)	**Dr Edward Raby** (ID Physician, WA)	**Ms Vanessa Nube** (Podiatrist, NSW)	**Ms Terry Swanson** (Wound Care Nurse, VIC)
Expert (Member)					**A/Prof Sara Jones** (Podiatrist, SA)	
Representative (Consumer)	**Ms Jane Cheney** (Consumer, VIC)	**Ms Jane Cheney** (Consumer, VIC)	**Ms Jane Cheney** (Consumer, VIC)	**Ms Jane Cheney** (Consumer, VIC)	**Ms Jane Cheney** (Consumer, VIC)	**Ms Jane Cheney** (Consumer, VIC)
Representative (Aboriginal & Torres Strait Islander)	**A/Prof James Charles** (Podiatrist, VIC)	**A/Prof James Charles** (Podiatrist, VIC)	**A/Prof James Charles** (Podiatrist, VIC)	**A/Prof James Charles** (Podiatrist, VIC)	**A/Prof James Charles** (Podiatrist, VIC)	**A/Prof James Charles** (Podiatrist, VIC)
Total members	**7**	**7**	**7**	**6**	**8**	**7**

^a^Expert: (Inter)national research and/or clinical practice diabetes-related foot disease (DFD) sub-field expert; Representative: Consumer or Aboriginal and Torres Strait Islander representative with expertise in DFD

*A/Prof* Associate Professor, *ID* Infectious Diseases, *NSW* New South Wales, *NT* Northern Territory, *Prof* Professor, *QLD* Queensland, *SA* South Australia, *TAS* Tasmania, *VIC* Victoria, *WA* Western Australia

Table 4 shows that 100 original recommendations (that addressed 51 clinical questions) were extracted from the 2019 IWGDF source guidelines. After categorisation into sub-fields the IWGDF recommendations were allocated as follows to each of the Australian expert panels: 16 to prevention, five to wound classification, 17 to PAD, 36 to infection, 13 to offloading, and 13 to wound healing. The IWGDF rated the quality (certainty) of evidence supporting these 100 recommendations as six (6%) having a high quality of evidence, 27 (27%) moderate, and 67 (67%) a low quality of evidence. The strength of recommendations were rated as 52 (52%) being a strong and 48 (48%) a weak recommendation. Table 5 shows that after all screening, assessment and/or re-evaluation of the 100 IWGDF source guideline recommendations, 98 Australian recommendations remained. The national panels rated the quality of evidence supporting these 98 Australian recommendations as three (3%) having a high quality of evidence, 24 (24%) moderate, and 71 (73%) as (very) low quality of evidence. The strength of recommendations were rated as 56 (57%) being a strong and 42 (43%) a weak recommendation.

**Table 4:** Summary of questions, recommendations, quality of evidence and strength of recommendations from the IWGDF guideline

Chapter	Questions	Recommendations	Quality of evidence^a^			Strength of Recommendation^b^	
			High	Moderate	Low	Strong	Weak
Prevention	11	16	2 (12%)	3 (19%)	11 (69%)	9 (56%)	7 (44%)
Wound classification	4	5	1 (20%)	3 (60%)	1 (20%)	3 (60%)	2 (40%)
PAD	8	17	0	3 (18%)	14 (82%)	17 (100%)	0
Infection	11	36	2 (6%)	13 (36%)	21 (58%)	13 (36%)	23 (64%)
Offloading	9	13	1 (8%)	2 (15%)	10 (77%)	5 (38%)	8 (62%)
Wound healing	8	13	0	3 (23%)	10 (77%)	5 (38%)	8 (62%)
TOTAL	51	100	6 (6%)	27 (27%)	67 (67%)	52 (52%)	48 (48%)

PAD: Peripheral artery disease

**a. Quality of evidence rating.** The quality of evidence is defined as the extent of the confidence that the estimates of an effect from a body of evidence are adequate to support a particular recommendation [26, 38, 45]. Quality of evidence can be rated as:

High = Typically, this is based on a body of evidence containing either: a) randomised trial(s) reporting similar effects with minimal risk of bias, inconsistency, indirectness, imprecision or publication bias &/or b) observational study(s) reporting similar very large effects, evidence of a dose response gradient and minimal confounding. Therefore, we are very confident that the true effect lies close to the estimate of the effect and further research is very unlikely to change our confidence in the estimate of effect [38, 45]

Moderate = Typically, this is based on a body of evidence containing either: a) randomised trial(s) reporting mostly similar effects, but with some serious risk of bias, inconsistency, indirectness, imprecision or publication bias, &/or b) observational study(s) reporting similar large effects with minimal confounding. Therefore, we are moderately confident that the true effect is likely to be close to the estimate of the effect, but there is also a possibility that it is substantially different and further research is likely to have an important impact on our confidence in the estimate of effect [38, 45]

Low = Typically, this is based on a body of evidence containing either: a) randomised trial(s) reporting some similar effects, but with very serious risk of bias, inconsistency, indirectness, imprecision or publication bias, &/or b) observational study(s) reporting similar effects, but with confounding [45]. Therefore, we have limited confidence that the true effect is likely to be close to the estimate of the effect, and there is a high possibility that it is substantially different and further research is very likely to have an important impact on our confidence in the estimate of effect [38, 45]

**b. Strength of recommendation ratings.** The strength of a recommendation is defined as the extent to which we can be confident that the desirable effects (i.e. benefits, such as improved health outcome, improved quality of life, decreased costs) of an intervention outweigh the undesirable effects (i.e. harms, such as adverse events, decreased quality of life, increased costs) [26, 30, 38]. The strength of a recommendation can be rated as:

Strong = Typically, this is based on a body of evidence, supplemented by expert opinion if limited evidence is available, that the desirable effects of an intervention considerably outweigh the undesirable effects for an intervention or vice versa. Therefore, we are highly confident of the balance between desirable and undesirable consequences and we make a strong recommendation for (desirable outweighs undesirable) or against (undesirable outweighs desirable) an intervention [30, 38]

Weak = Typically, this is based on a body of evidence, supplemented by expert opinion if limited evidence is available, that the desirable effects of an intervention may outweigh the undesirable effects for an intervention or vice versa. Therefore, we are less confident of the balance between desirable and undesirable effects and we make a weak recommendation for (desirable outweighs undesirable) or against (undesirable outweighs desirable) an intervention [30, 38].

**Table 5:** Summary of questions, recommendations, quality of evidence and strength of recommendations from the new Australian guidelines

Chapter	Questions	Recommendations	Quality of evidence^a^				Strength of Recommendation^b^	
			High	Moderate	Low	Very Low	Strong	Weak
Prevention	11	15	0	2 (13%)	13 (87%)	0	9 (60%)	6 (40%)
Wound classification	4	5	1 (20%)	3 (60%)	1 (20%)	0	2 (40%)	3 (30%)
PAD	8	17	0	3 (18%)	14 (82%)	0	17 (100%)	0
Infection	11	35	2 (6%)	12 (34%)	20 (57%)	1 (3%)	21 (60%)	14 (40%)
Offloading	9	13	0	1 (8%)	9 (69%)	3 (23%)	4 (31%)	9 (69%)
Wound healing	8	13	0	3 (23%)	10 (77%)	0	3 (23%)	10 (77%)
TOTAL	51	98	3 (3%)	24 (24%)	67 (68%)	4 (4%)	56 (57%)	42 (43%)

PAD: Peripheral artery disease

**a. Quality of evidence rating.** The quality of evidence is defined as the extent of the confidence that the estimates of an effect from a body of evidence are adequate to support a particular recommendation [26, 38, 45]. Quality of evidence can be rated as:

High = Typically, this is based on a body of evidence containing either: a) randomised trial(s) reporting similar effects with minimal risk of bias, inconsistency, indirectness, imprecision or publication bias &/or b) observational study(s) reporting similar very large effects, evidence of a dose response gradient and minimal confounding. Therefore, we are very confident that the true effect lies close to the estimate of the effect and further research is very unlikely to change our confidence in the estimate of effect [38, 45]

Moderate = Typically, this is based on a body of evidence containing either: a) randomised trial(s) reporting mostly similar effects, but with some serious risk of bias, inconsistency, indirectness, imprecision or publication bias, &/or b) observational study(s) reporting similar large effects with minimal confounding. Therefore, we are moderately confident that the true effect is likely to be close to the estimate of the effect, but there is also a possibility that it is substantially different and further research is likely to have an important impact on our confidence in the estimate of effect [38, 45]

Low = Typically, this is based on a body of evidence containing either: a) randomised trial(s) reporting some similar effects, but with very serious risk of bias, inconsistency, indirectness, imprecision or publication bias, &/or b) observational study(s) reporting similar effects, but with confounding [45]. Therefore, we have limited confidence that the true effect is likely to be close to the estimate of the effect, and there is a high possibility that it is substantially different and further research is very likely to have an important impact on our confidence in the estimate of effect [38, 45]

Very Low = Typically, this is based on a body of evidence containing either: a) observational study(s) reporting similar effects, but with confounding, &/or expert opinion [45]. Therefore, we have very limited confidence that the true effect is likely to be close to the estimate of the effect, and there is a very high possibility that it is substantially different and further research is most likely to have an important impact on our confidence in the estimate of effect [38, 45]

**b. Strength of recommendation ratings.** The strength of a recommendation is defined as the extent to which we can be confident that the desirable effects (i.e. benefits, such as improved health outcome, improved quality of life, decreased costs) of an intervention outweigh the undesirable effects (i.e. harms, such as adverse events, decreased quality of life, increased costs) [26, 30, 38]. The strength of a recommendation can be rated as:

Strong = Typically, this is based on a body of evidence, supplemented by expert opinion if limited evidence is available, that the desirable effects of an intervention considerably outweigh the undesirable effects for an intervention or vice versa. Therefore, we are highly confident of the balance between desirable and undesirable consequences and we make a strong recommendation for (desirable outweighs undesirable) or against (undesirable outweighs desirable) an intervention [30, 38]

Weak = Typically, this is based on a body of evidence, supplemented by expert opinion if limited evidence is available, that the desirable effects of an intervention may outweigh the undesirable effects for an intervention or vice versa. Therefore, we are less confident of the balance between desirable and undesirable effects and we make a weak recommendation for (desirable outweighs undesirable) or against (undesirable outweighs desirable) an intervention [30, 38].

In summary, after screening all 100 IWGDF source guideline recommendations the panels deemed 68 (68%) were acceptable and applicable to the Australian health context and were adopted without further assessment. The other 32 (32%) were judged to have unsure (or no) acceptability and/or applicability in the Australian health context and required full assessment, including nine in offloading, eight in prevention, seven in infection, four in wound healing interventions, three in wound classification, and one in PAD. After full assessment of those 32 recommendations, three more were adopted, 27 adapted and two excluded for the Australian context. Therefore, of 100 IWGDF source guideline recommendations, 71 (71%) were adopted, 27 (27%) adapted and 2 (2%) excluded in the Australian guidelines. The six individual sub-field guidelines report details of all decisions for each sub-field [31–36].

### Drafting recommendations and rationale

Overall, of those 27 adapted recommendations, nine were in offloading, six in prevention, four in infection, four in wound healing interventions, three in wound classification and one in PAD. The main reasons for adapting recommendations included: 20 had wording changed to be considered acceptable in Australia; ten had quality of evidence changed; four had strength of the recommendation changed; four had wording changed to be considered feasible in Australia; and/or, one had the direction for the balance of effects changed. Of the two recommendations excluded, one was in the prevention and one the infection guideline [31, 34]. The prevention recommendation was excluded because the panel had substantial differences in judgements compared with the IWGDF judgements for the desirable effects, balance of effects and quality of supporting evidence for a recommendation concerning “performing foot and mobility-related exercises with the aim of reducing risk factors of ulceration” (IWGDF Prevention Recommendation 14) [31]. The infection recommendation was excluded because the panel had substantial differences in judgements compared with the IWGDF judgements for the balance of effects and quality of supporting evidence due to the inclusion of evidence for a heterogeneous population for a recommendation concerning to “treat diabetes-related foot osteomyelitis with antibiotic therapy for no longer than 6 weeks” (IWGDF Infection Recommendation 23A) [34].

### Developing guideline manuscripts

In total, alongside this guideline development protocol manuscript, six sub-field guideline manuscripts were drafted (prevention, wound classification, PAD, infection, offloading, and wound healing interventions). Collectively these form the new *2021 Australian evidence-based guidelines for diabetes-related foot disease*. Detailed reasoning behind all 98 recommendations included in the guidelines are described in those six sub-field guideline manuscripts. Therefore, we refer all Australian health professionals caring for people with, or at risk of, DFD, to the *2021 Australian evidence-based guidelines for diabetes-related foot disease* [31–36].

### External consultation and approval of guideline manuscripts

Table 6 displays the aggregated summary public consultation survey response findings. A total of 47 responses (27 individual and 20 organisational responses) were received across the six sub-field guidelines; with prevention and offloading receiving the most responses with 19 and 14 respectively [31–36]. In summary, > 85% of respondents (strongly) agreed (with < 10% disagreeing) that: there was a need for new Australia DFD guidelines; the methods used to develop the guidelines were appropriate, objective and transparent; the recommendations made were clear; they agreed with the recommendations made; and the recommendations if implemented should produce more benefits than harms, better use of resources, and would be acceptable to people with DFD. However, to implement the recommendations, 60% (strongly) agreed they may require some reorganisation of services, 55% agreed they may be technically challenging and 39% agreed they may be too expensive. Overall, > 80% (strongly) agreed (with < 5% disagreeing) that the guidelines should be approved as the new Australian guidelines, they would be supported by the majority of their colleagues and they would use or encourage their use in practice. Additionally, all de-identified feedback comments received during public consultation and each panel’s responses to each comment were collated and posted on the DFA website.

**Table 6:** Summary public consultation survey responses across all six guidelines (*n* = 47)

No.	Item	n	Strongly Agree	Agree	Neither Agree or Disagree	Disagree	Strongly Disagree
Background							
1	You are involved with the care of patients for whom this draft Australian guideline is relevant.	47	31 (66.0%)	9 (19.1%)	7 (14.9%)	0	0
2	There is a need for a new Australian guideline in this population.	47	23 (48.9%)	20 (42.6%)	3 (6.4%)	1 (2.1%)	0
3	The rationale for developing a new Australian guideline on this topic is clear in this draft guideline.	47	29 (61.7%)	17 (36.2%)	1 (2.1%)	0	0
Methodology							
4	I agree with the overall methodology used to develop this draft Australian guideline.	47	20 (42.6%)	23 (48.9%)	4 (8.5%)	0	0
5	The search strategy used to identify international guidelines on which this draft Australian guideline was based is relevant and complete	47	19 (40.4%)	23 (48.9%)	4 (8.5%)	1 (2.1%)	0
6	The methods used to determine the suitability of identified international source guidelines upon which this draft Australian guideline were based were robust.	47	20 (42.6%)	21 (44.7%)	6 (12.8%)	0	0
7	I agree with the methods used within this draft Australian guideline to interpret the available evidence on this topic.	47	18 (38.3%)	24 (51.1%)	5 (10.6%)	0	0
8	The methods used to decide which recommendations to adopt, adapt or exclude for the Australian context were objective and transparent.	47	17 (36.2%)	27 (57.4%)	3 (6.4%)	0	0
Recommendations							
9	The recommendations in this draft Australian guideline are clear.	46	22 (47.8%)	19 (41.3%)	4 (8.7%)	1 (2.2%)	0
10	I agree with the recommendations in this draft Australian guideline as stated.	46	14 (30.4%)	24 (52.2%)	5 (10.9%)	3 (6.5%)	0
11	The recommendations are suitable for people living with diabetes-related foot disease.	46	15 (32.6%)	26 (56.5%)	3 (6.5%)	2 (4.3%)	0
12	The recommendations are too rigid to apply for people living with diabetes-related foot disease.	46	3 (6.5%)	4 (8.7%)	8 (17.4%)	27 (58.7%)	6 (13.0%)
13	The recommendations reflect a more effective approach to improving patient outcomes than is current practice.	46	10 (21.7%)	13 (28.3%)	17 (37.0%)	6 (13.0%)	0
14	When applied, the recommendations should produce more benefits than harms for people living with diabetes-related foot disease.	46	19 (41.3%)	22 (47.8%)	4 (8.7%)	1 (2.2%)	0
15	When applied, the recommendations should result in better use of resources than current practice allows.	46	16 (34.8%)	13 (28.3%)	13 (28.3%)	4 (8.7%)	0
16	I would feel comfortable if people living with diabetes-related foot disease received the care recommended in this draft Australian guideline.	46	21 (45.7%)	20 (43.5%)	5 (10.9%)	0	0
Implementation of recommendations							
17	To apply the draft Australian guideline may require reorganisation of services/care.	45	9 (20.0%)	18 (40.0%)	12 (26.7%)	5 (11.1%)	1 (2.2%)
18	To apply the draft Australian guideline may be technically challenging.	45	6 (13.3%)	19 (42.2%)	14 (31.1%)	4 (8.9%)	2 4.4%)
19	The draft Australian guideline may be too expensive to apply.	45	8 (17.8%)	5 (11.1%)	15 (33.3%)	13 (28.9%)	4 (8.9%)
20	The draft Australian guideline presents options that will likely be acceptable to people living with diabetes-related foot disease.	45	10 (22.2%)	29 (64.4%)	2 (4.4%)	4 (8.9%)	0
Final thoughts							
21	This draft guideline should be approved as the new Australian guideline.	45	19 (42.2%)	18 (40.0%)	6 (13.3%)	2 (4.4%)	0
22	This draft Australian guideline would be supported by the majority of my colleagues.	45	17 (37.8%)	22 (48.9%)	6 (13.3%)	0	0
23	If this draft guideline was to be approved as the new Australian guideline, I would use or encourage their use in practice.	45	23 (51.1%)	18 (40.0%)	3 (6.7%)	1 (2.2%)	0

Based on the collated public consultation feedback, the guideline manuscripts were finally revised and approved by the relevant national panel and authors. The final manuscripts were endorsed as the *2021 Australian evidence-based guidelines for diabetes-related foot disease* by ten national peak bodies including the Australian Podiatry Association, Wounds Australia, Australian and New Zealand Society for Vascular Surgery, Australasian Society for Infectious Diseases, Australian Orthotic Prosthetic Association, Pedorthic Association of Australia, Australian Advanced Practicing Podiatrists - High Risk Foot Group, Australian Aboriginal and Torres Strait Islander Diabetes-related Foot Complications Program, the Australian Diabetes Society and DFA. The final endorsed guidelines, including pathways, are displayed in full on the DFA website https://www.diabetesfeetaustralia.org/new-guidelines/ [31–36], registered on the Australian Clinical Practice Guidelines register [14] and were submitted to peer-reviewed journals for publication. Finally, the authors completed the ADAPTE Checklist for Adapted Guideline Content to ensure all guideline elements had been completed [17] (Supplementary Material Table S3).

## Discussion

For the first time in a decade we have developed new Australian evidence-based guidelines for diabetes-related foot disease by systematically adapting suitable high-quality international (IWGDF) source guidelines to the Australian health context. Of the 100 original IWGDF recommendations, 71 were adopted, 27 adapted and two excluded for use in Australia across six guideline manuscripts. These guidelines have now been endorsed by ten national peak bodies to serve as the new national guidelines and multidisciplinary best practice standards for the provision of DFD care within Australia [31–36].

There are some marked differences between these new 2021 Australian DFD guidelines [31–36] and the previous 2011 Australian DFD guideline [10] which perhaps begin to illustrate the strength and limitations of the new 2021 guidelines [11]. A significant strength of the previous 2011 guideline was that it received considerable Australian Government funding to specifically develop a national DFD guideline from scratch (“de novo*”*) [10]. This funding enabled systematic reviews to be specifically constructed and performed for the Australian context by methodologist organisations, augmented by the expert opinion of a 13 member DFD expert panel, and all adhering to NHMRC recommendations of the time [10]. Whereas, due to a scarcity of funding available for these new 2021 guidelines, we had to adapt suitable high-quality international guidelines (IWGDF) to the Australian health context [31–36]. This limited the content of these new 2021 guidelines to only those recommendations covered by the IWGDF source guidelines. However, to try and minimise these limitations we followed various NHMRC recommended processes for adapting such suitable international source guidelines to the Australian health context, including using best practice tools from ADAPTE, AGREE II and GRADE systems [13, 17, 18, 24, 26, 27], and six national expert sub-field panels consisting of 30 (inter)national experts, including consumer and Aboriginal and Torres Strait Islander experts [15, 16]. Lastly, a limitation of both guidelines was the delay between when the systematic reviews were performed and when the recommendations were published in the guidelines; 2009 systematic reviews for the previous 2011 guideline [10] and 2019 systematic reviews [38–44] for these new 2021 guidelines [31–36].

Adapting the IWGDF guidelines may also be seen as a strength for the new 2021 guidelines due to the breadth of coverage and methodological quality provided by the IWGDF source guidelines [38–44], plus the ability to address some minor IWGDF guideline methodological limitations identified by our AGREE II quality assessments (Table 1) [11]. In terms of coverage, these 2021 Australian guidelines specifically outline 98 recommendations across six individual sub-field guidelines [38–44], compared with 25 recommendations in one overarching previous 2011 guideline (that partially covered four sub-fields of prevention, offloading, wound classification and wound healing interventions, but did not cover PAD or infection) [10]. In terms of quality, we not only used the IWGDF guidelines rated as having high overall quality in our AGREE II quality assessments [24], we then followed the gold standard ADAPTE framework as the methodological steps to adapt the IWGDF guidelines [17], and the contemporary international gold standard GRADE system for synthesising and grading both the quality of evidence and strength of each recommendation [18, 26–30, 45]. The previous Australian guideline graded only the quality of evidence via the previous NHMRC grades of recommendation, which were the national gold standard of the time [10]. Furthermore, in terms of addressing identified minor methodological IWGDF limitations, unlike IWGDF we engaged both consumer and Aboriginal and Torres Strait Islander experts in all panel decisions, plus, asked all panels to provide specific considerations for the implementation of all recommendations in Aboriginal and Torres Strait Islander and geographically remote populations. Additionally, we provided the opportunity for wide public consultation from the Australian DFD community, revised accordingly and developed user-friendly clinical pathways for all guidelines to optimise the ease of uptake of all recommendations for multi-disciplinary health professionals following a formal pathway development process.

In future iterations of these guidelines, we would hope to either align an updated adaptation of new Australian guidelines more closely with the development of the new 2023 IWGDF source guidelines [38] or obtain the significant funding to develop the next DFD guideline de novo [13]. An avenue for such funding of de novo guidelines may be that of developing living guidelines as recently published for other Australian diabetes-related complications [46]. If such funding does become available to develop new guidelines de novo we suggest that additional sub-fields are also considered and addressed, such as Charcot foot and inpatient DFD care.

## Conclusion

New Australian DFD guidelines have been developed for the first time in 10 years using best practice methodology. Over 30 national experts systematically evaluated an existing high-quality international IWGDF DFD source guideline and made necessary adaptations to be applicable and acceptable to Australian clinical contexts. A minimum of a month-long public consultation process occurred with feedback transparently incorporated where appropriate. These new DFD guidelines are endorsed by ten national peak bodies. The authors strongly urge all Australian health professionals from all disciplines caring for people at risk of or with DFD to implement all new guideline recommendations that accompany this guideline development protocol to help reduce the large national burden of DFD in Australia.

## Supplementary Information

**Additional file 1: Table S1:** Customised tool for assessing a guidelines suitability to adopt or adapt. **Table S2:** Public consultation survey example. **Table S3:** The completed ADAPTE Checklist of adapted guideline content. **Figure S1:** Customised ADAPTE Evaluation of acceptability and applicability form. **Figure S2:** Customised GRADE Evidence to Decision template.

## Data Availability

Data sharing is not applicable to this article as no datasets containing patient information were generated or analysed during the current study.

## Abbrevations

ADS: Australian Diabetes Society
AGREE II: Appraisal of Guidelines for Research and Evaluation II Instrument
DFA: Diabetes Feet Australia
DFD: Diabetes-related foot disease
EtD: Evidence to Decision
GRADE: Grading of Recommendations Assessment, Development and Evaluation
IWGDF: International Working Group on the Diabetic Foot
NHMRC: National Health and Medical Research Council
PAD: Peripheral artery disease
PIPOH: Population, Intervention, Professions, Outcome, Healthcare context
PN: Peripheral neuropathy
