# Supplementary material for: Guidelines development protocol and findings: part of the 2021 Australian evidence-based guidelines for diabetes-related foot disease
Source: J Foot Ankle Res. 2022 Apr 19;15:28. doi: 10.1186/s13047-022-00533-8 (PMC9017044; doi:10.1186/s13047-022-00533-8)

**Supplementary Material for:**

**Guidelines development protocol and findings: Part of the 2021 Australian evidence-based guidelines for diabetes-related foot disease**

Lazzarini PA, Raspovic A, Prentice J, Commons RJ, Fitridge RA, Charles J, Cheney J, Purcell N, Twigg SM, on behalf of the Australian Diabetes-related Foot Disease Guidelines & Pathways Project

**Table of contents Page**

**TABLES**  2

**Table S1:** Customised tool for assessing a guidelines suitability to adopt or adapt 2

**Table S2:** Public consultation survey example 5

**Table S3:** The completed ADAPTE Checklist of adapted guideline content 9

**FIGURES**  11

**Figure S1:** Customised ADAPTE Evaluation of acceptability and applicability form 11

**Figure S2:** Customised GRADE Evidence to Decision template 15

# **TABLES**

**Table S1:** Customised tool for assessing a guidelines suitability to adopt or adapt.

***Based on the NHMRC Guidelines for guidelines: Adopt, adapt or start from scratch.***

**Name of Assessor: __________________________________________________________**

**Date of Assessment: _________________________________________________________**

**Email of Assessor: __________________________________________________________**

**Instructions**

Please complete Table 1 over the page to assess the suitability for the guidelines and associated guideline documents (systematic reviews and appendices) attached.

This table has been customised from Table 2 of the NHMRC Guidelines for guidelines: Adopt, adapt or start from scratch at the end of this document. Please refer to Table 2 for context as it provides an overview of the factors to consider when you assess a guideline’s suitability to adopt or adapt, as well as suggestions for how to check this information on suitability (under the ‘Approach’ column in Table 2).

To help provide further discrimination with answers the same 7-point Likert scale used in the AGREE II tool (i.e. 1=Strongly disagree to 7=Strongly agree). has been incorporated into the customised table. Thus, please circle (or underline or **bold**) the number to denote your answer for each item in Table 1 that you assess as most appropriate after reading the IWGDF Guidelines and associated guideline documents provided for each of the 22 items. Finally, don’t forget to complete the scores at the end of each domain/section and the total score at the end.

**Now please complete the table over page**

| Item | Question | Strongly Disagree |  |  |  |  |  | Strongly Agree |
| --- | --- | --- | --- | --- | --- | --- | --- | --- |
|  | **Relevance** |  |  |  |  |  |  |  |
| 1 | Is the clinical or public health context similar to Australia? | 1 | 2 | 3 | 4 | 5 | 6 | 7 |
| 2 | Are the population, intended users and settings comparable? | 1 | 2 | 3 | 4 | 5 | 6 | 7 |
| 3 | Are the recommended interventions available in Australia? | 1 | 2 | 3 | 4 | 5 | 6 | 7 |
| 4 | Are the guideline questions relevant in the new (Australian) context? | 1 | 2 | 3 | 4 | 5 | 6 | 7 |
| 5 | Do the values and preferences considered in the guideline reflect the new (Australian) context? | 1 | 2 | 3 | 4 | 5 | 6 | 7 |
| 6 | Are relevant outcomes used? | 1 | 2 | 3 | 4 | 5 | 6 | 7 |
|  | **Score (please add scores for 6 items)** |  |  |  |  |  |  |  |
|  | ***Currency** | **Strongly**  **Agree** |  |  |  |  |  | **Strongly Disagree** |
| 7 | When was the evidence review conducted (i.e. final literature search date)? | **Date:** |  |  |  |  |  |  |
| 8 | Is the evidence contained out of date? | 1 | 2 | 3 | 4 | 5 | 6 | 7 |
| 9 | Are new studies’ findings conducted since the review likely to change the evidence? | 1 | 2 | 3 | 4 | 5 | 6 | 7 |
| 10 | Has new evidence superseded the information contained in the recommendations? | 1 | 2 | 3 | 4 | 5 | 6 | 7 |
| 11 | Does new evidence contradict the recommendations? | 1 | 2 | 3 | 4 | 5 | 6 | 7 |
|  | **Score (please add scores for 4 applicable items)** |  |  |  |  |  |  |  |
|  | **Trustworthiness** | **Strongly Disagree** |  |  |  |  |  | **Strongly Agree** |
| 12 | Is there a detailed description of the development process? | 1 | 2 | 3 | 4 | 5 | 6 | 7 |
| 13 | Were conflicts of interest declared and managed? | 1 | 2 | 3 | 4 | 5 | 6 | 7 |
| 14 | Was a grading system used for the recommendations? | 1 | 2 | 3 | 4 | 5 | 6 | 7 |
| 15 | Are the evidence tables clearly laid out and accurate? | 1 | 2 | 3 | 4 | 5 | 6 | 7 |
| 16 | Was the evidence review systematic and well-documented? | 1 | 2 | 3 | 4 | 5 | 6 | 7 |
|  | **Score (please add scores for 5 items)** |  |  |  |  |  |  |  |
|  | **Access to evidence** |  |  |  |  |  |  |  |
| 17 | Are the tables detailing the source evidence (e.g. GRADE Evidence to Decision tables) available? | 1 | 2 | 3 | 4 | 5 | 6 | 7 |
| 18 | Can permission be sought to use these tables? | 1 | 2 | 3 | 4 | 5 | 6 | 7 |
|  | **Score (please add scores for 5 items)** |  |  |  |  |  |  |  |
|  | **Implementability** |  |  |  |  |  |  |  |
| 19 | Is information is provided in the guideline to assist implementation? | 1 | 2 | 3 | 4 | 5 | 6 | 7 |
| 20 | Are steps taken to improve the guideline’s implementability? | 1 | 2 | 3 | 4 | 5 | 6 | 7 |
|  | **Score (please add scores for 5 items)** |  |  |  |  |  |  |  |
|  | **Acceptability** |  |  |  |  |  |  |  |
| 21 | Are the recommendations acceptable? | 1 | 2 | 3 | 4 | 5 | 6 | 7 |
| 22 | Do the recommendations relate to current practice? | 1 | 2 | 3 | 4 | 5 | 6 | 7 |
|  | **Score (please add scores for 5 items)** |  |  |  |  |  |  |  |
|  | **TOTAL SCORE (please add scores for all 21 applicable items)** |  |  |  |  |  |  |  |
|  | **Comments (**please add any comments you feel necessary to provide context on scores) |  |  |  |  |  |  |  |

*Please note only the Currency items answers change direction from 1= Strongly Disagree to instead 1= Strongly Agree

**Table S2:** Public consultation survey example


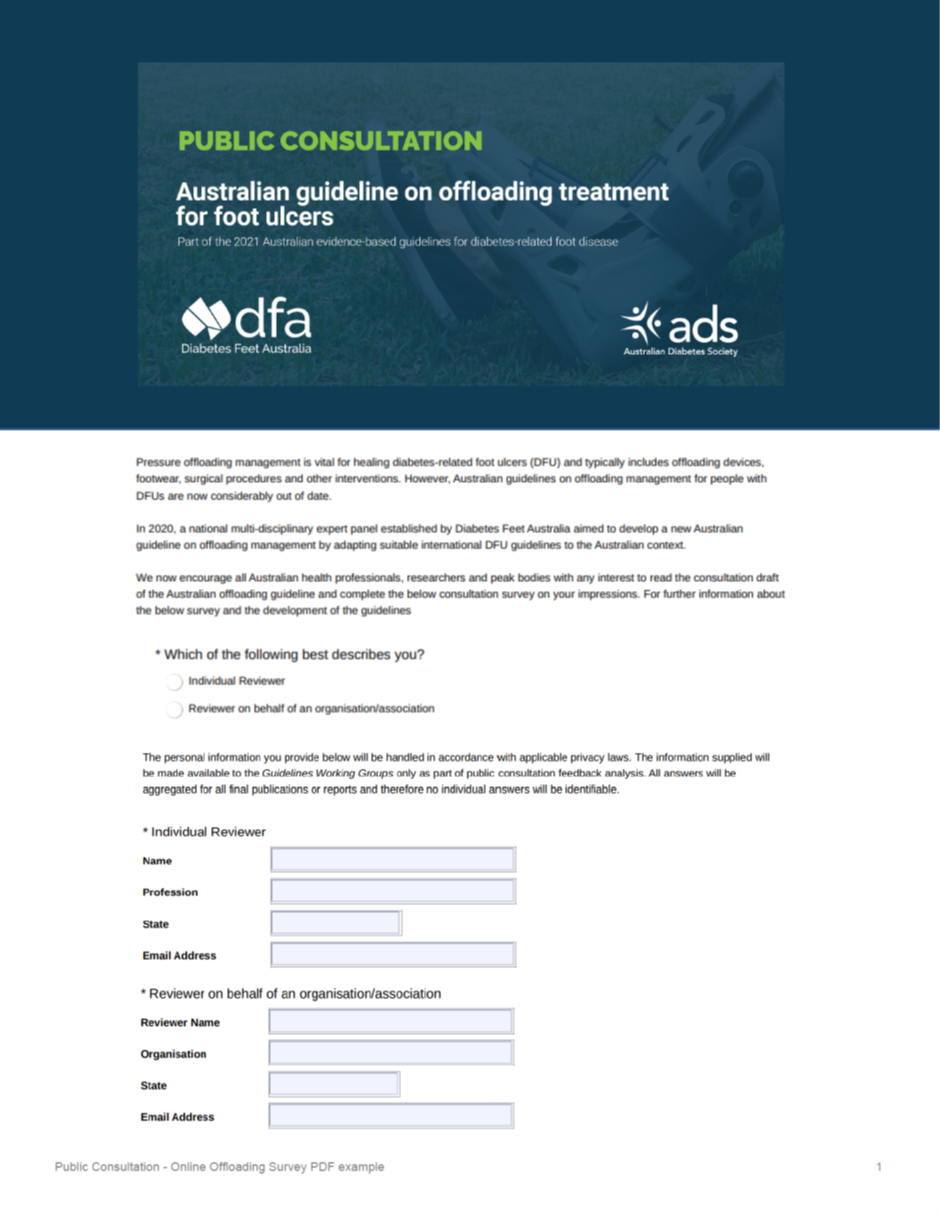


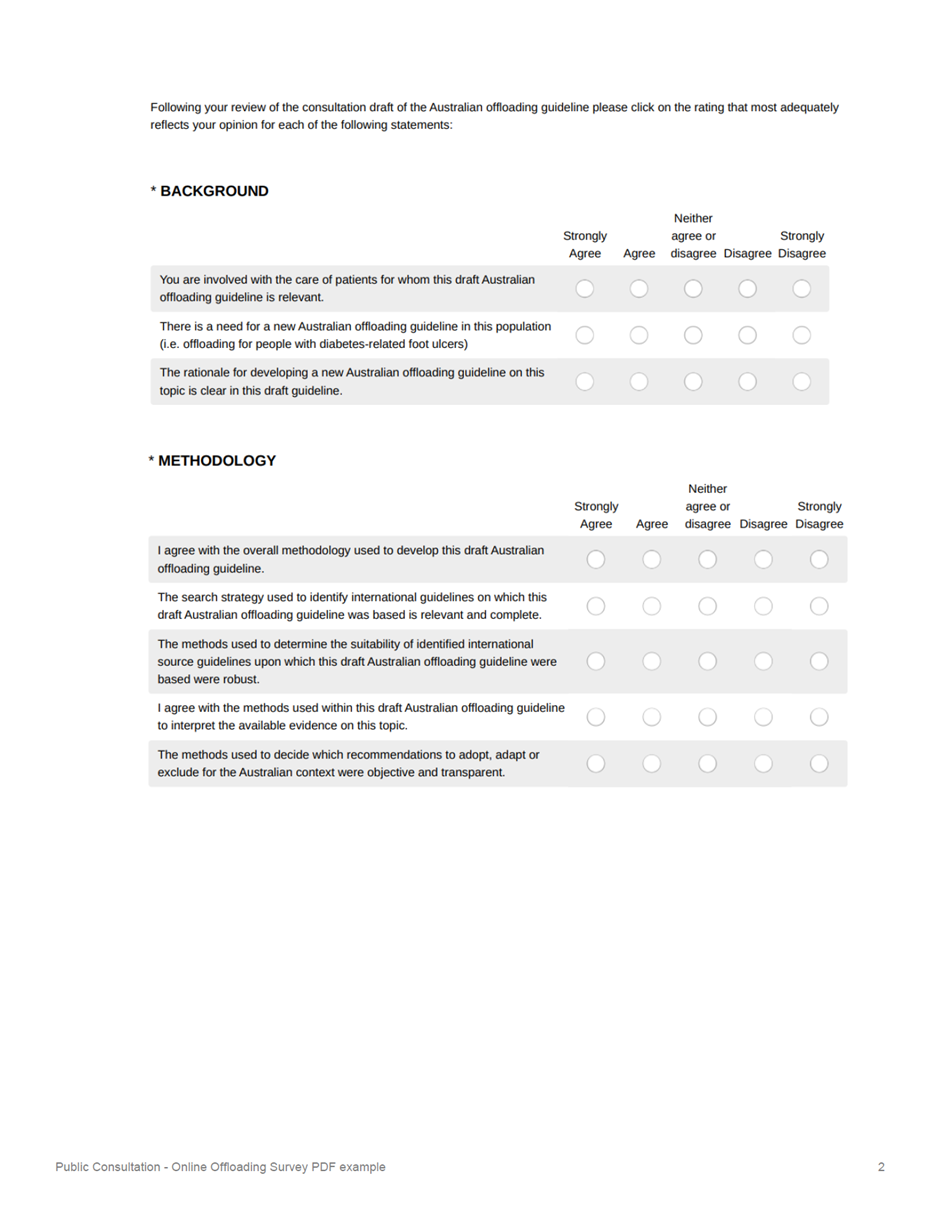


**
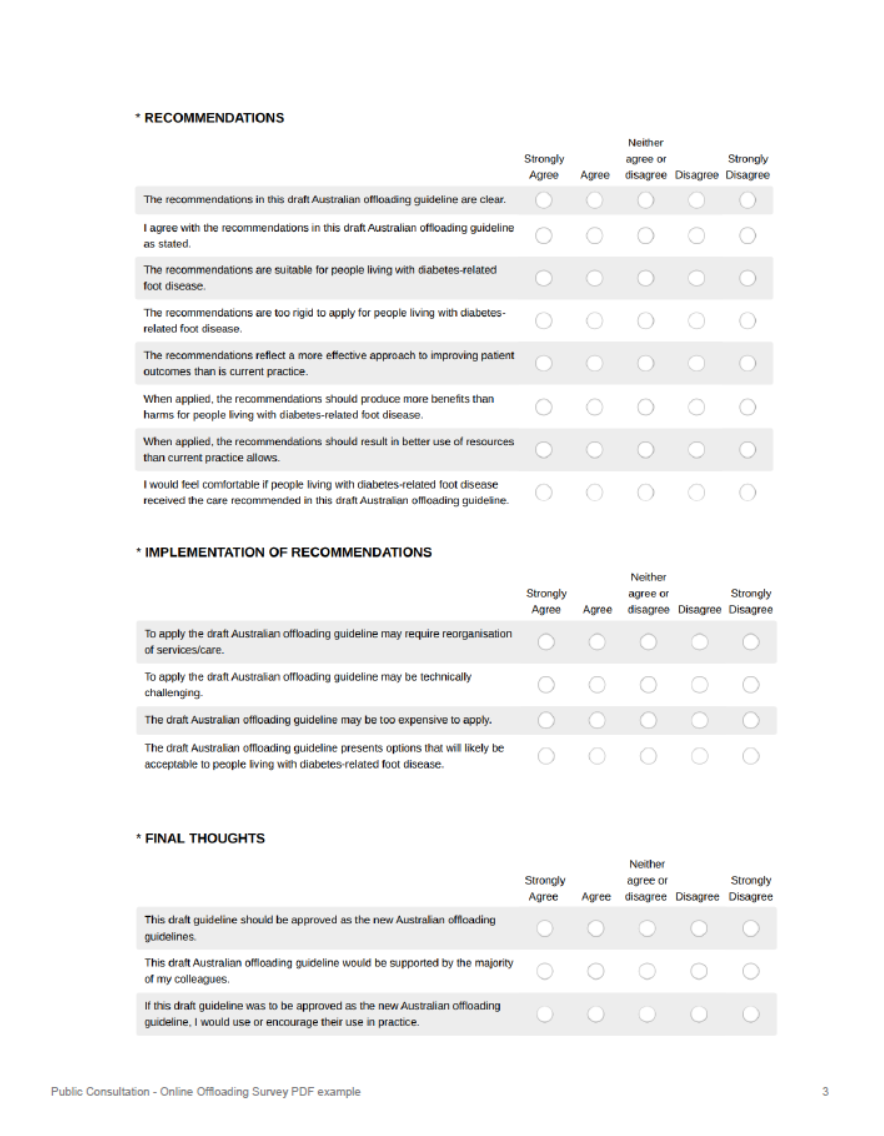
**

**
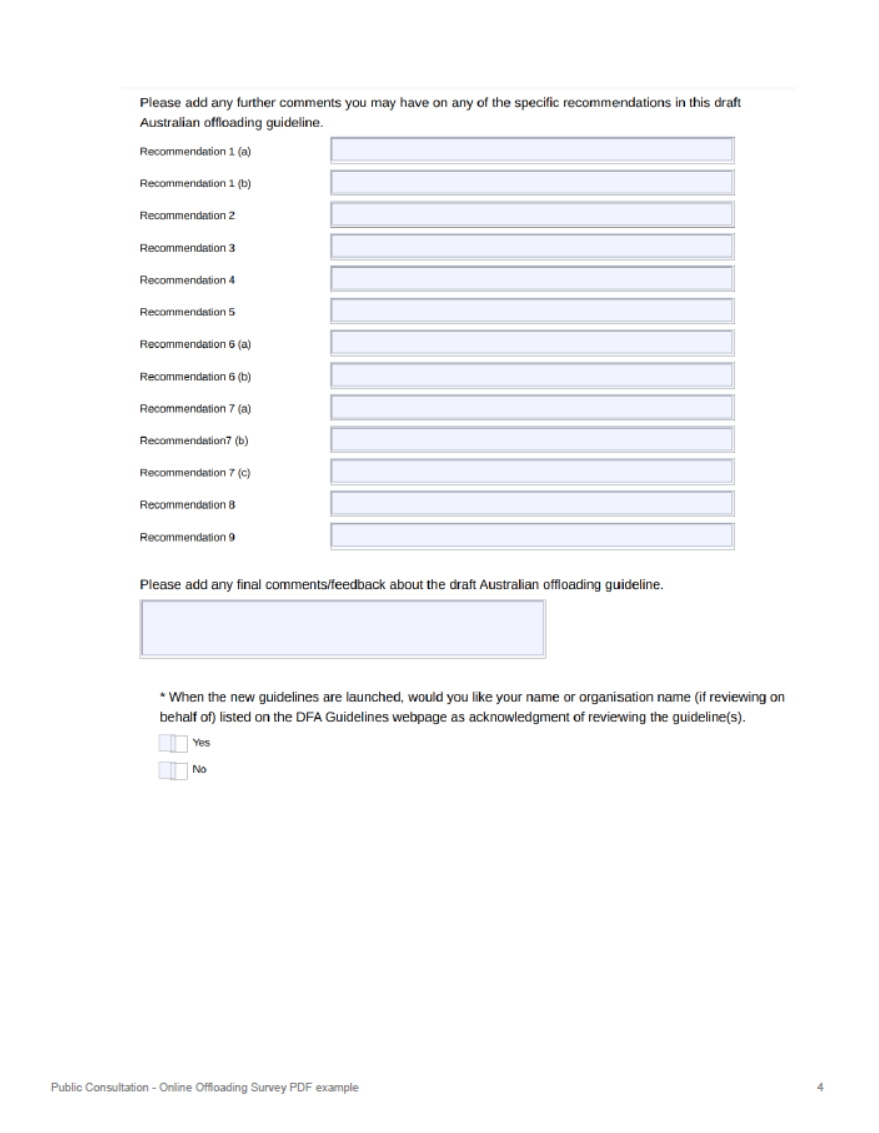
**

**Table S3:** The completed ADAPTE Checklist of adapted guideline content

| Item  No. | Guideline section | Completed (and where in documents) |
| --- | --- | --- |
| 1 | Overview material |  |
|  | 1. Structured abstract including:  - Guidelines release date - Status (original, adapted, revised, updated) - Print and electronic sources | Yes. Abstract: Page 3-4. |
|  | 1. Adapter and source guideline developer | Yes. Abstract: Page 3-4. |
| 2 | Introduction and background | Yes. Background: Page 5. |
| 3 | Scope and purpose | Yes. Methods i) Defining scope: Page 7. |
| 4 | Target audience of the guideline | Yes. Background: Page 5;  Methods i) Defining the scope of the guidelines: Page 7;  Methods ii) Identifying potential international source guidelines: Page 7-8. |
| 5 | Health questions | Yes. Methods iv) a. Recommendations were categorised into six DFD sub-fields: Page 10;  Recommendation sections of all individual guidelines. |
| 6 | Recommendations   - Risk and benefits associated with the recommendations - Specific circumstances under with to perform the recommendation - Strength of recommendation (if assigned) | Yes. Methods iv) Deciding which recommendations to adopt, adapt and exclude: Pages 10-13;  Methods v) Drafting recommendations and reasoning for those recommendations: Pages 14-16;  Recommendation sections of all individual guidelines. |
| 7 | Supporting evidence and information for the recommendations   - Panel rationale behind the recommendations - Presentation of additional evidence - How and why existing recommendations were modified | Yes. Methods iv) Deciding which recommendations to adopt, adapt and exclude: Pages 10-13;  Methods v) Drafting recommendations and reasoning for those recommendations: Pages 14-16;  Recommendation sections of all individual guidelines. |
| 8 | External review and consultation process   - Who was asked to review the guideline - What process was followed - Discussion of feedback - Feedback incorporated into the final document | Yes. Methods vii) External consultation and approval of guideline manuscripts: Page 17-18;  Results: External consultation and approval of guideline manuscripts: Page 17-18.  Table 6: Page 39-40;  Results sections of all individual guidelines; Guideline Response documents for all individual guidelines on the guideline website: <https://www.diabetesfeetaustralia.org/new-guidelines/> |
| 9 | Plan for scheduled review and update | Yes. Methods vii) External consultation and approval of guideline manuscripts: Page 17-18. |
| 10 | Algorithm or summary document | Yes. Methods viii) Developing clinical pathways to aide implementation into practice: Page 16;  Figure 1 of all individual guidelines. |
| 11 | Implementation considerations | Yes. Methods v) Drafting recommendations and reasoning for those recommendations: Pages 14-16;  Recommendation sections of all individual guidelines. |
| 12 | Glossary (for unfamiliar terms) | Yes. Glossary sections of individual guidelines (where required). |
| 13 | References of all material used in creating the guideline | Yes. Reference sections of all individual guidelines. |
| 14 | Acknowledgement of source guideline developers and permission granted (where necessary) | Yes. Acknowledgements section: Page 30. |
| 15 | List of panel members and their credentials, declarations of conflict of interest | Yes. Background: Page 5;  Methods iv) b. National expert panels were convened for each sub-field: Page 10-11;  Results Deciding which recommendations to adopt, adapt or exclude: Page 10-13;  Table 3: Page 36;  Competing interests: Page 28-29;  Competing interests section of all individual guidelines;  Panel member biographers and declarations of conflicts of interest on the guideline website: <https://www.diabetesfeetaustralia.org/new-guidelines/> |
| 16 | List of funding sources | Yes. Funding: Page 29. |
| 17 | Appendix describing adaptation process including:   - Guideline search and retrieval including list of guidelines and whether they were included/excluded, with rationale - Guideline assessments including a summary of results for each assessment (including AGREE domain score) - Decision process followed by panel - Results and decisions of each evaluation | Yes. Methods ii) Identifying potential source guidelines: Page 7-8;  Results Identifying potential international source guidelines: Page 19; Figure 1: Page 31;  Methods iii) Determining suitable international source guidelines to adapt: Pages 8-10;  Results: Determining suitable international source guidelines to adapt: Pages 19-20;  Tables 1-2: Page 32-35;  Results and Recommendation sections of individual guidelines. |

# **FIGURES**

**Figure S1:** Customised ADAPTE Evaluation of acceptability and applicability form


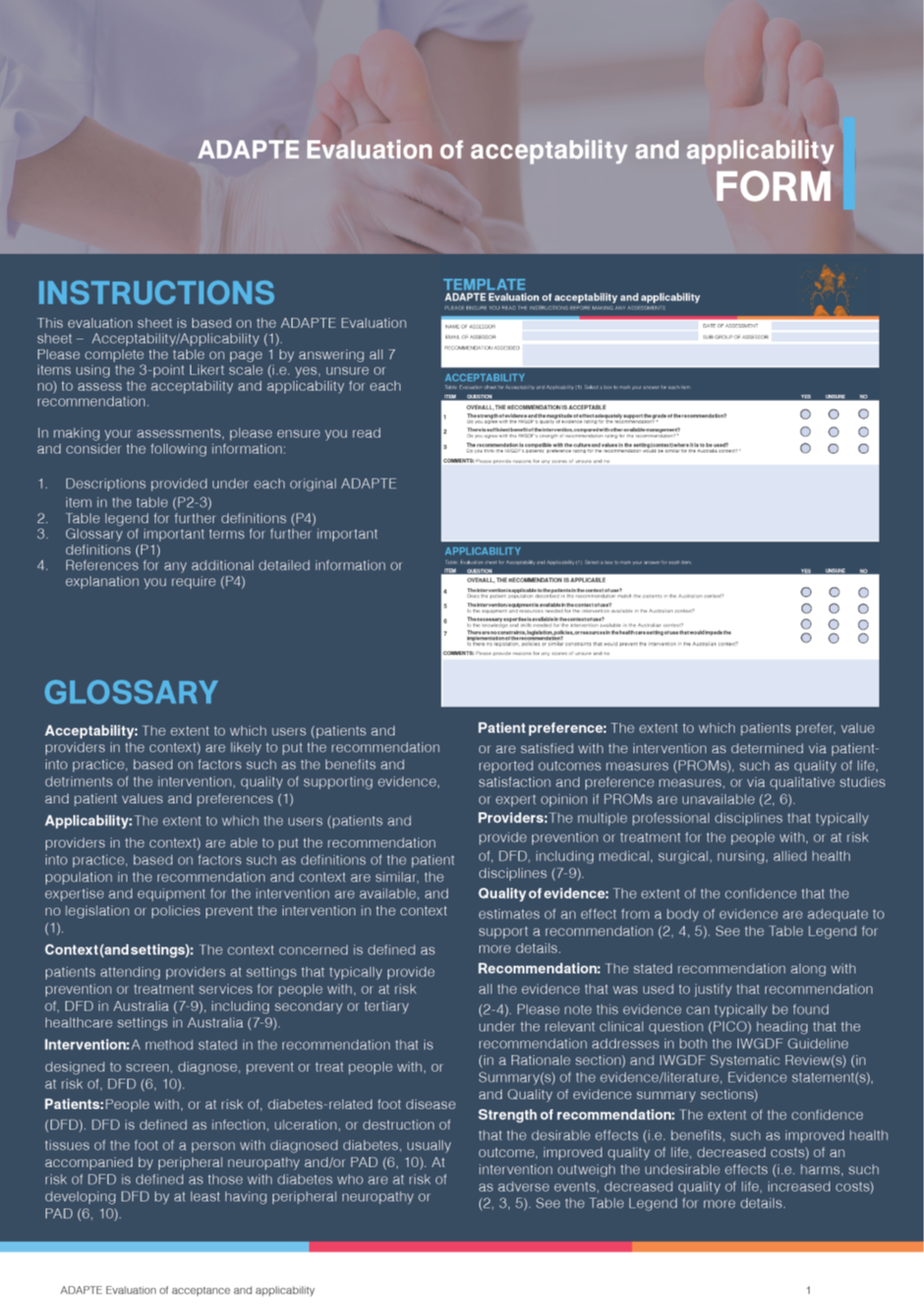


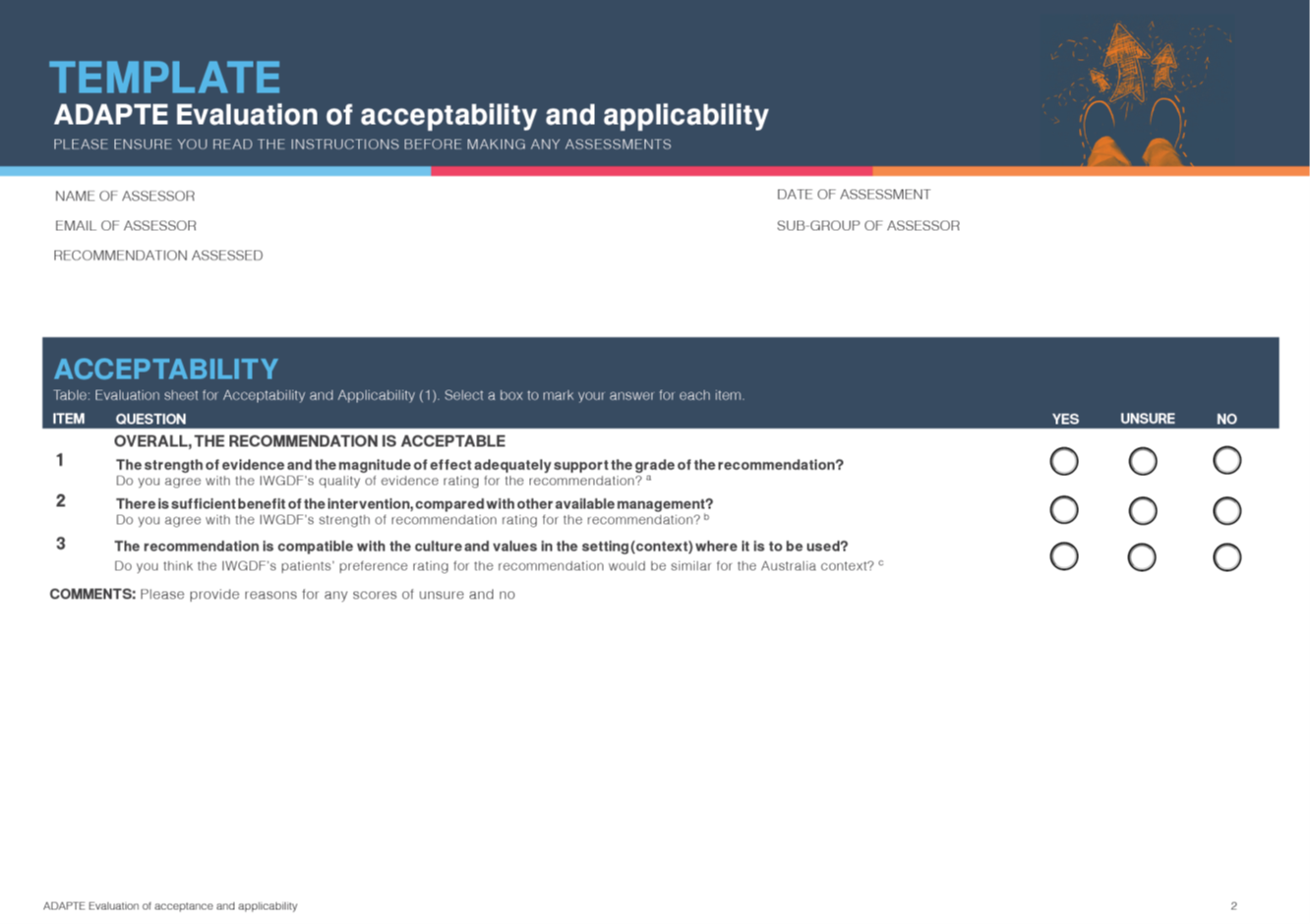


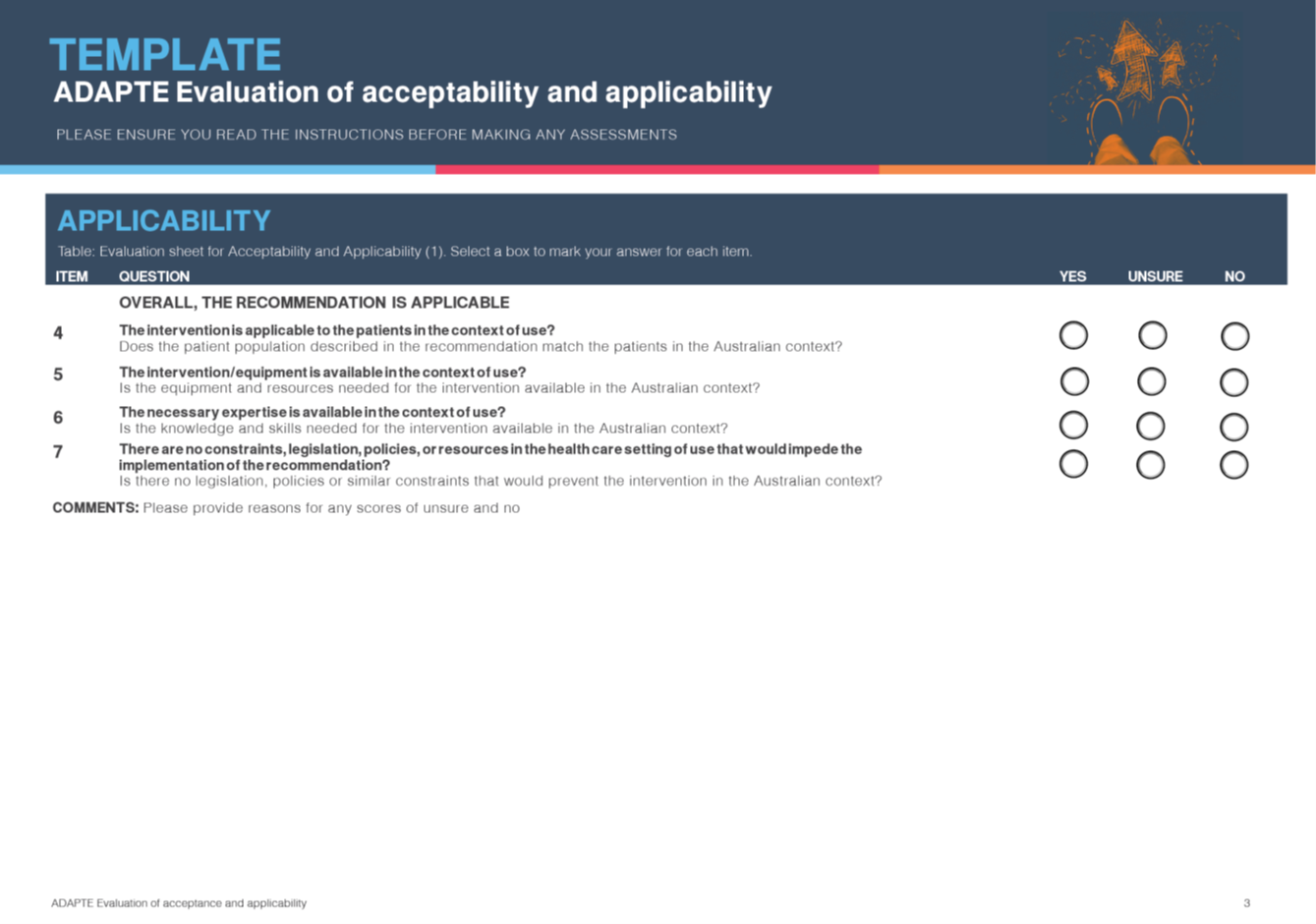


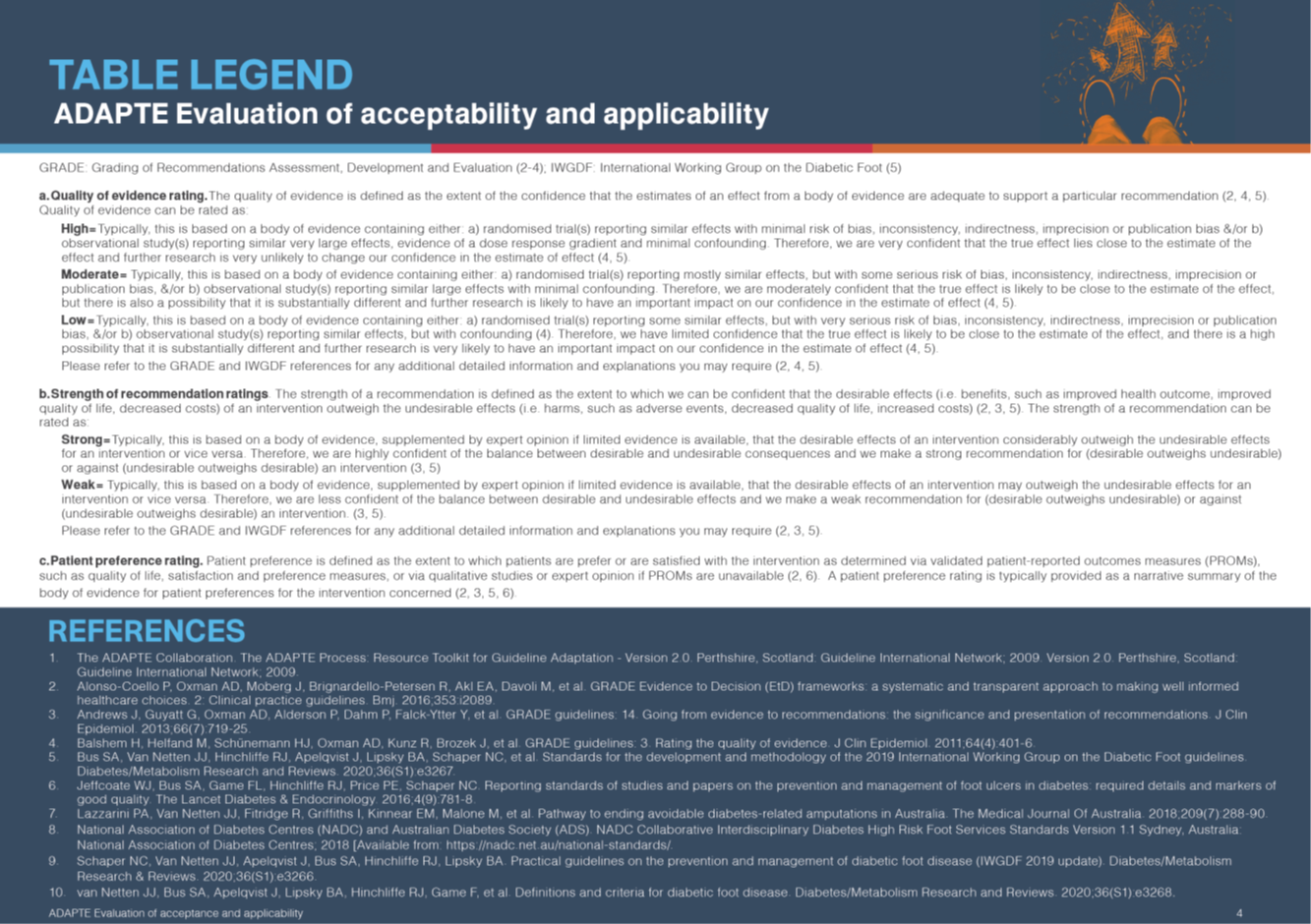


**Figure S2:** Customised GRADE Evidence to Decision template


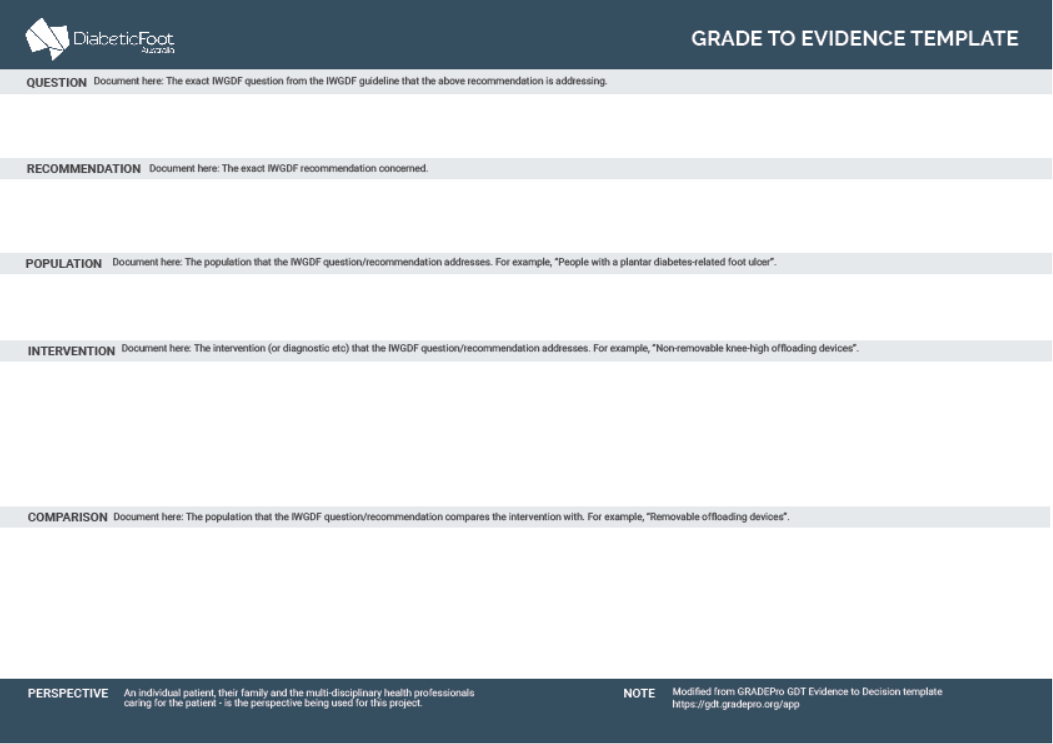


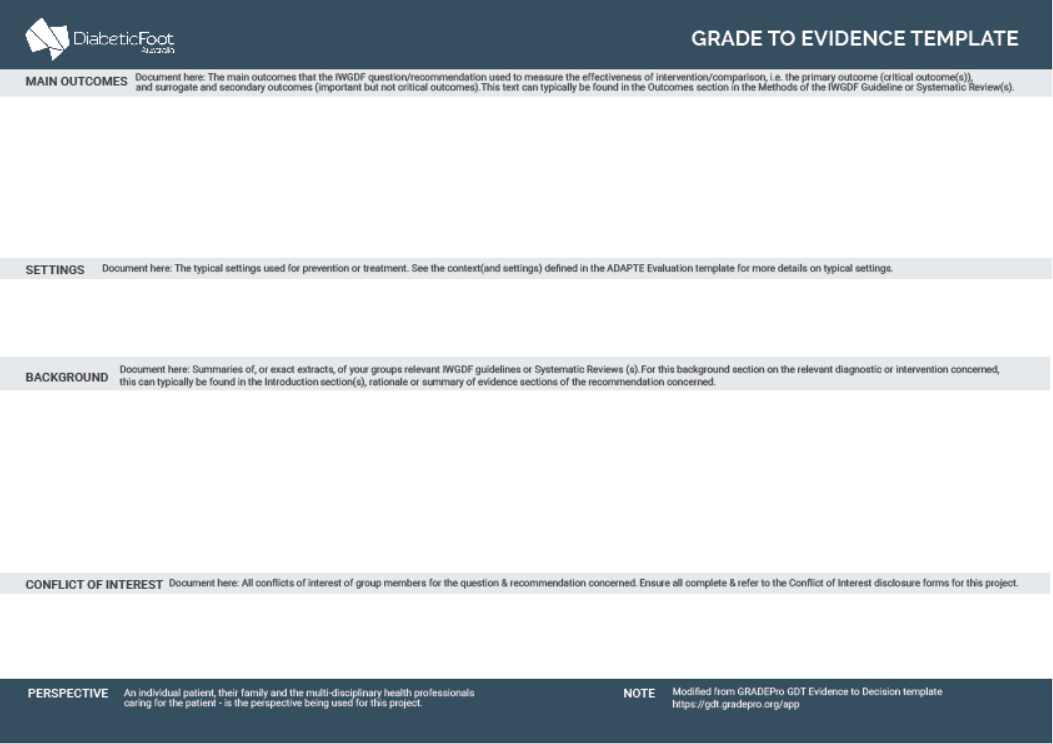


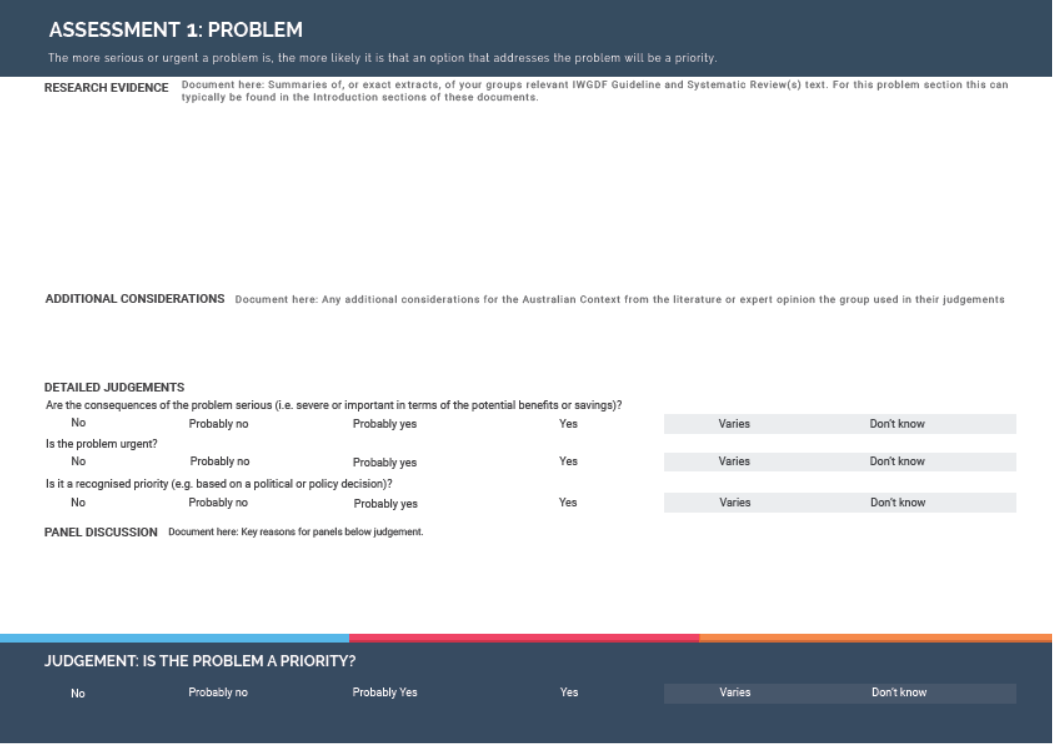


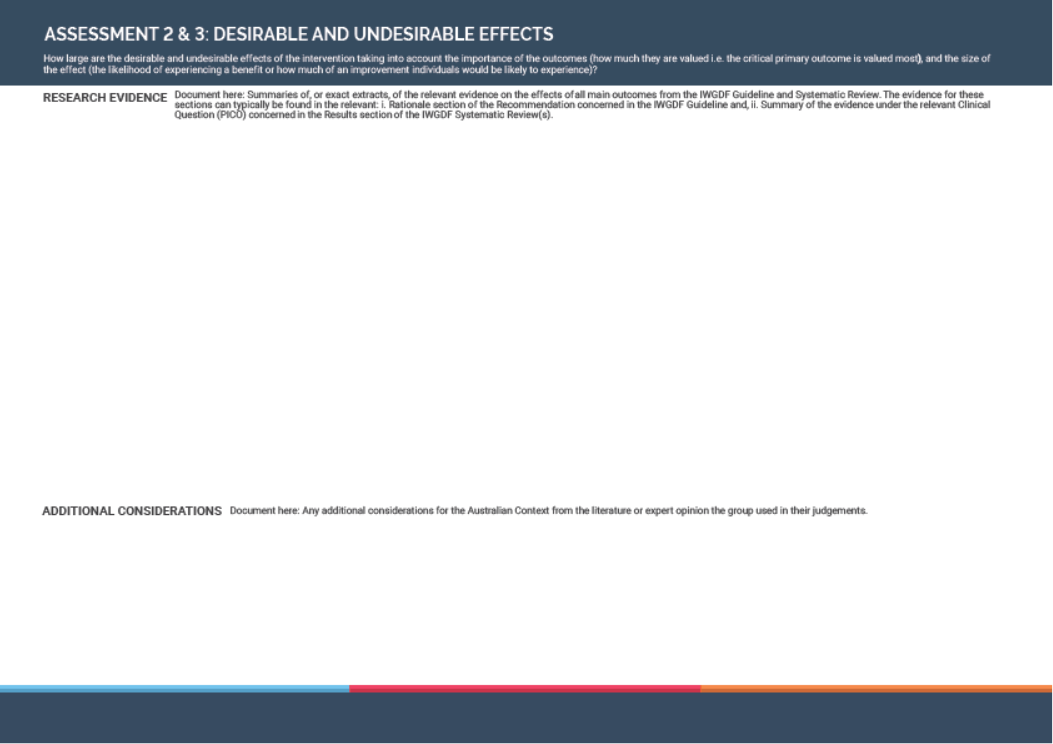


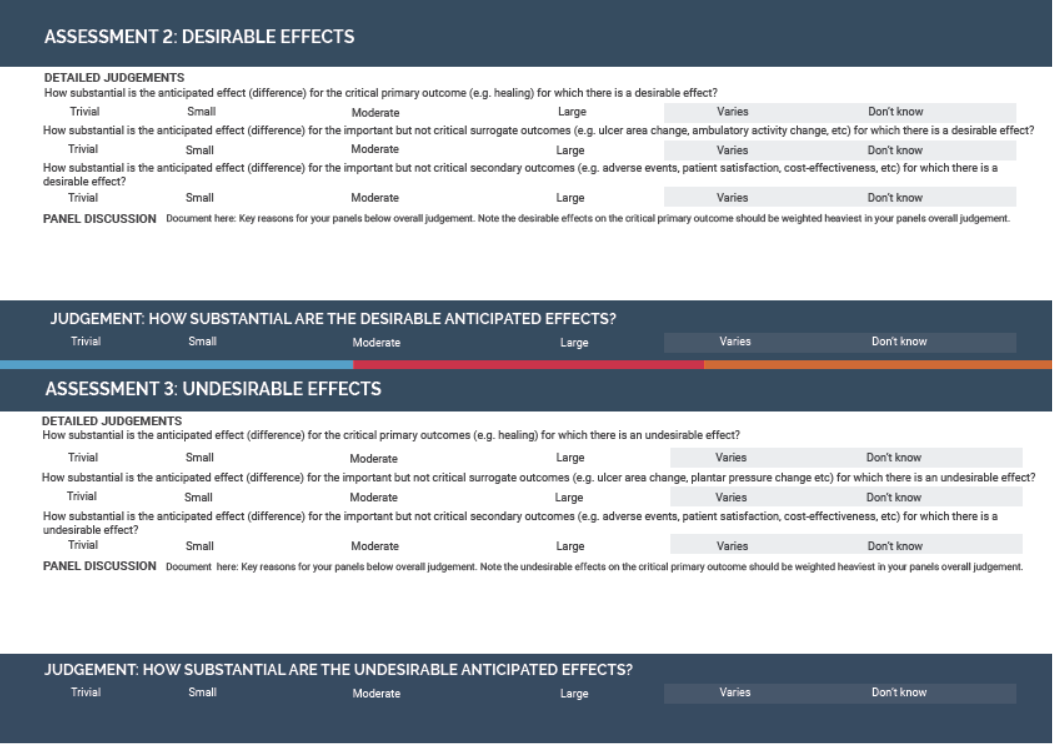


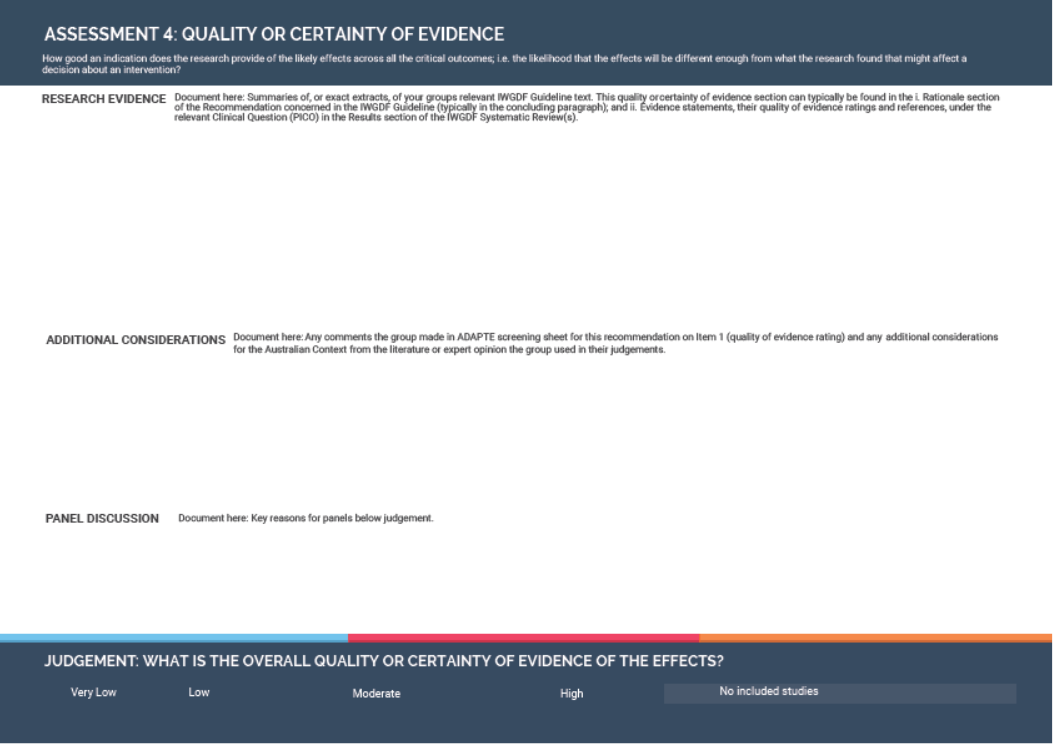


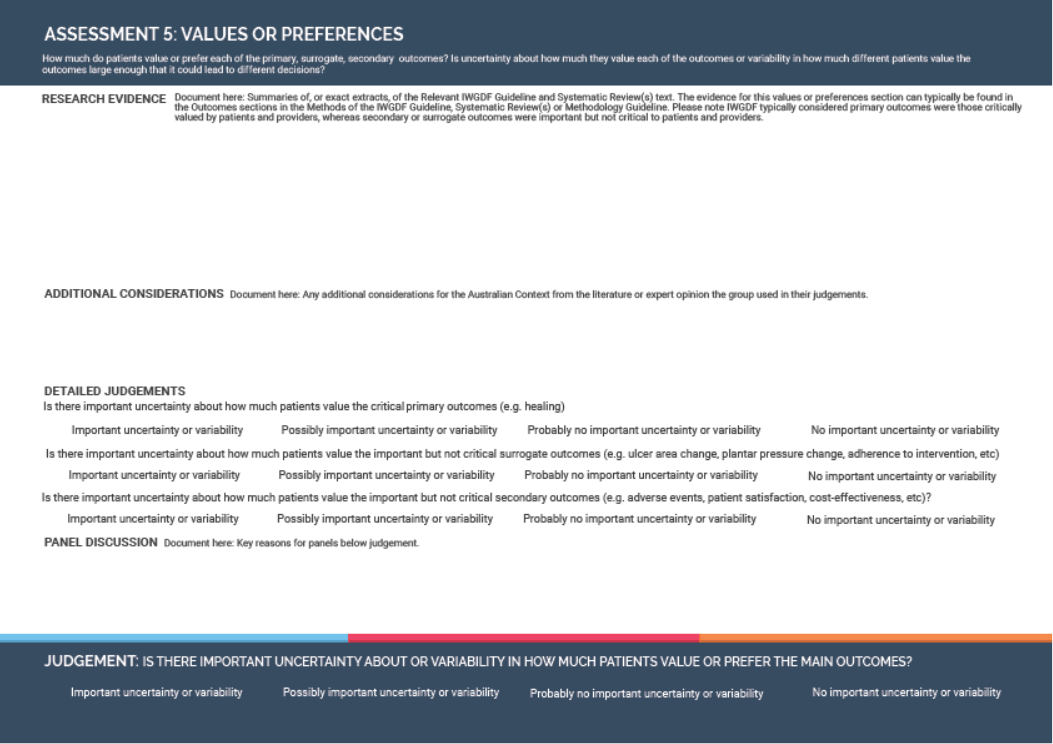


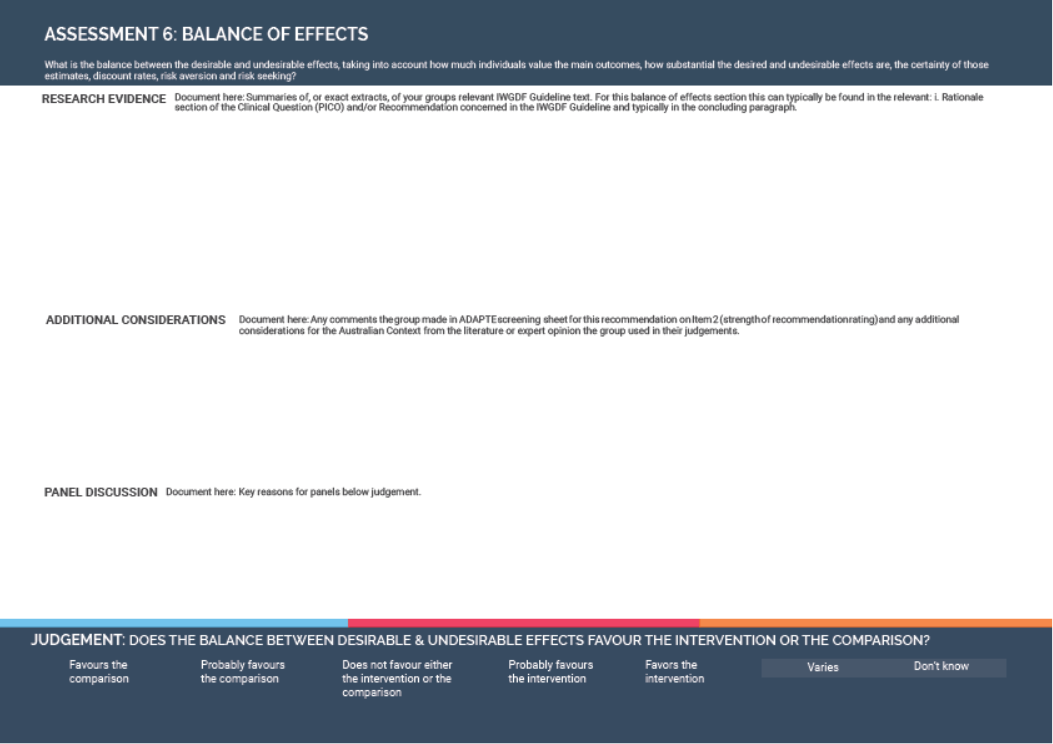


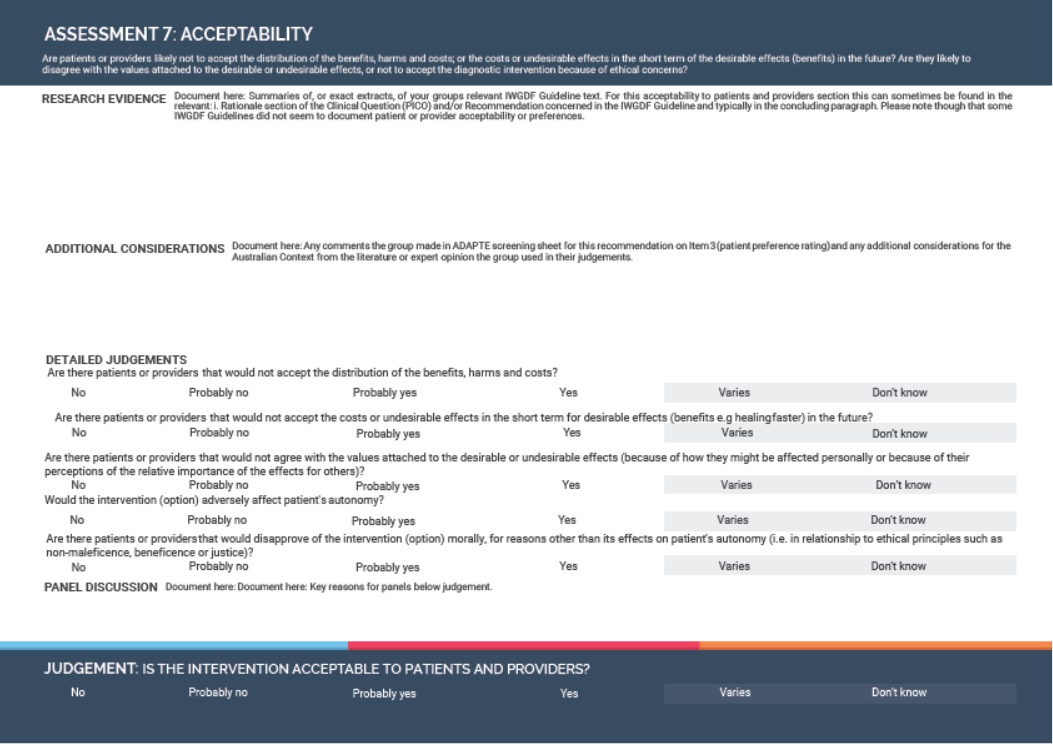


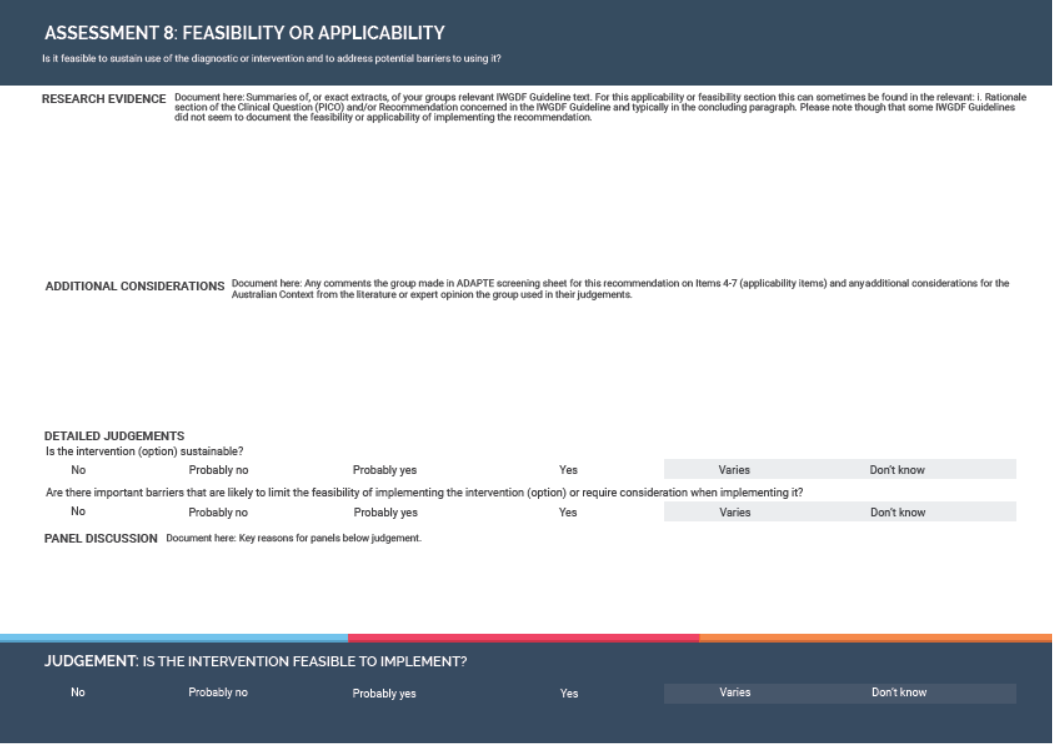


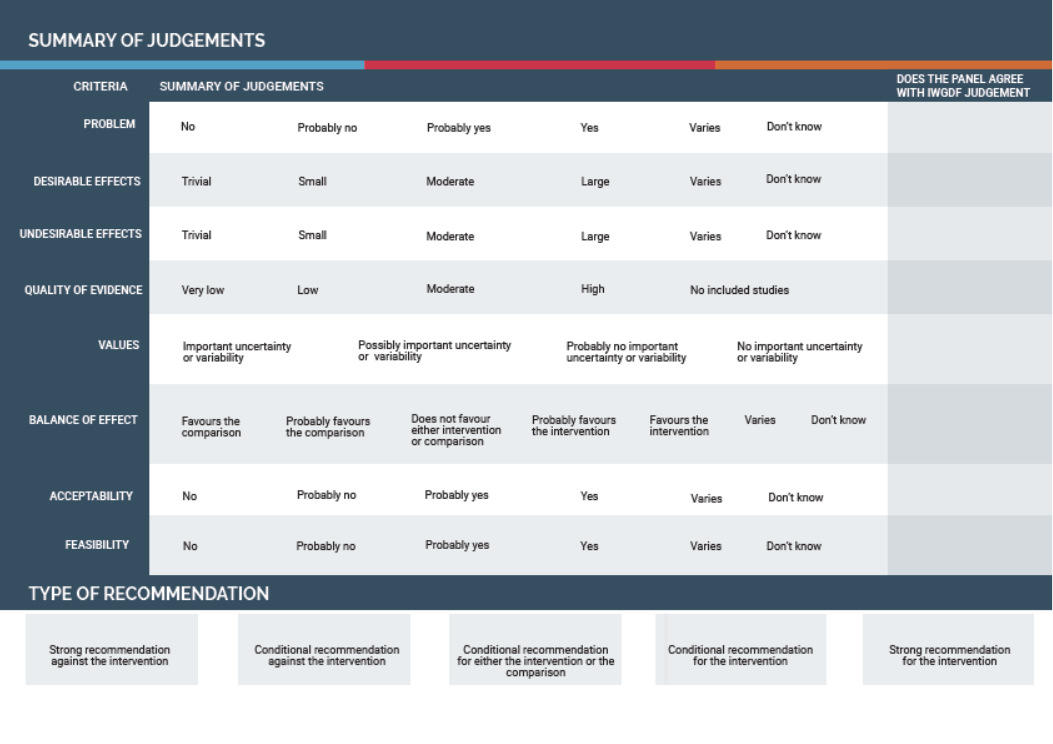


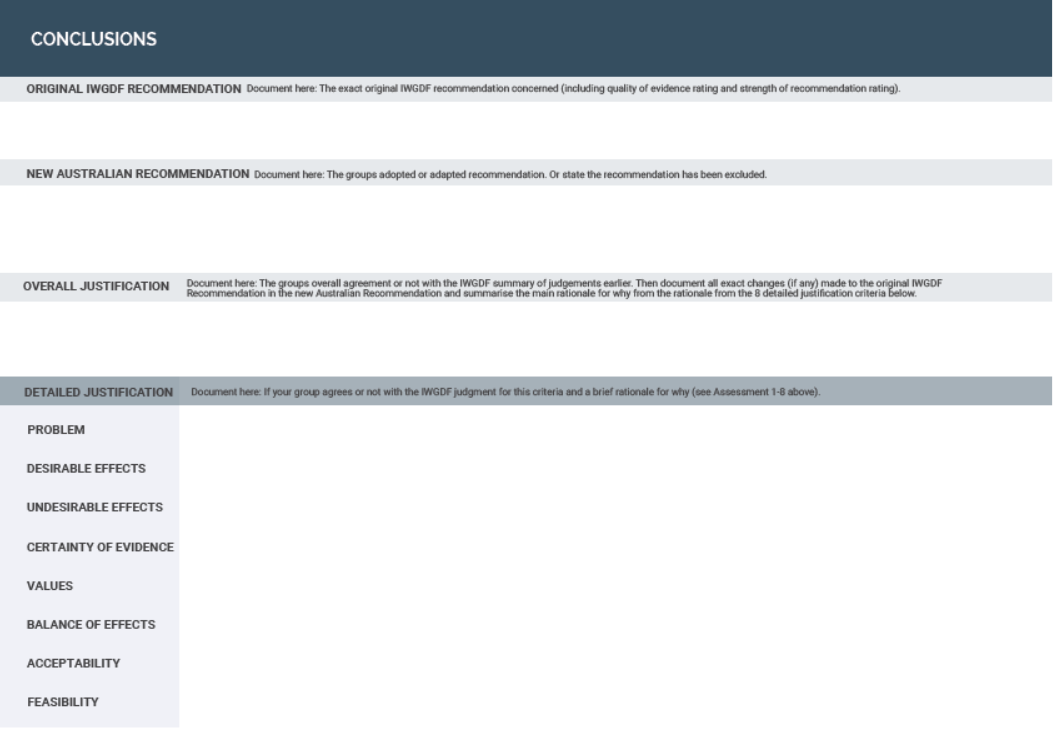


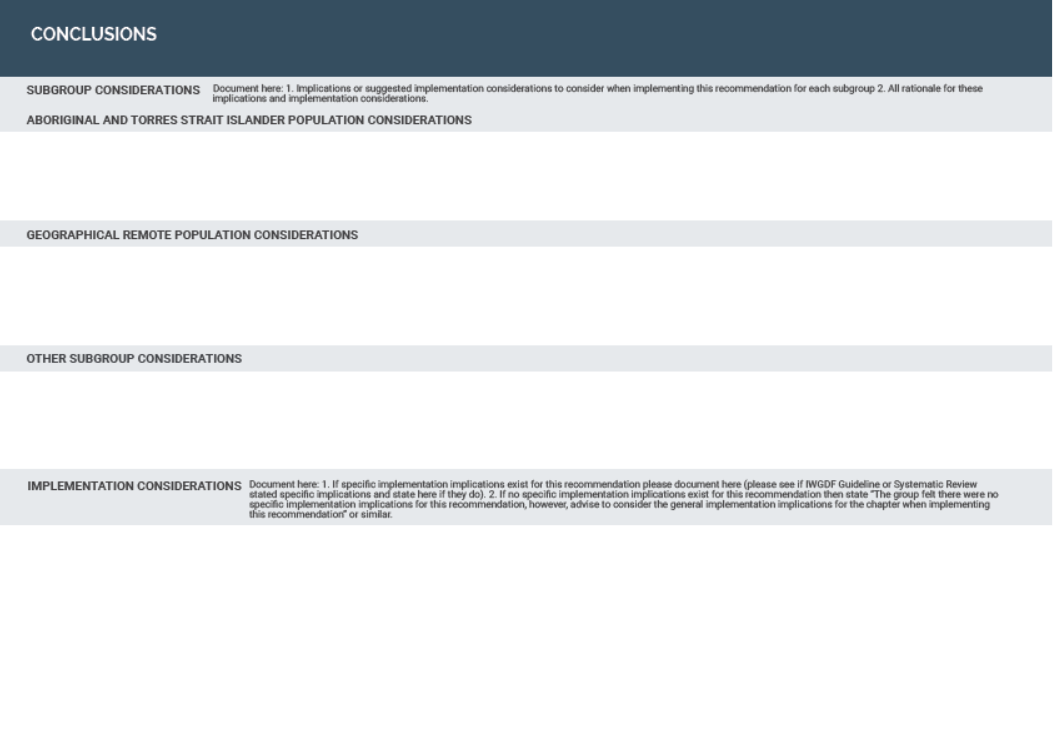


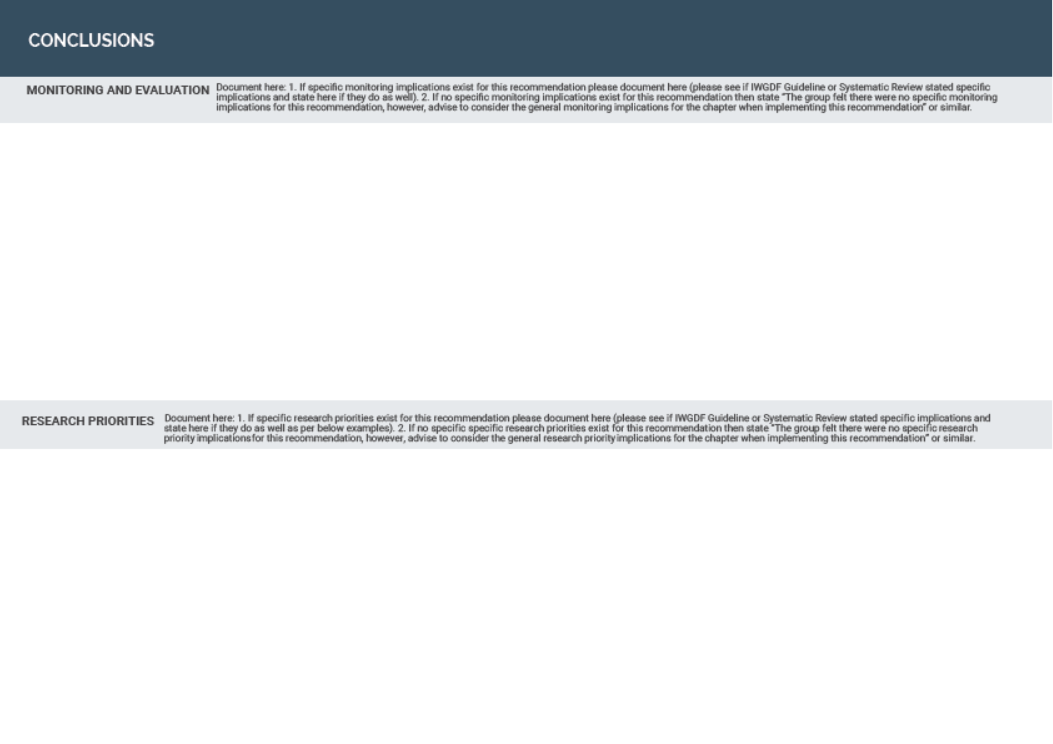

Supplement: Supplementary file 1 — Additional file 1: Table S1: Customised tool for assessing a guidelines suitability to adopt or adapt. Table S2: Public consultation survey example. Table S3: The completed ADAPTE Checklist of adapted guideline content. Figure S1: Customised ADAPTE Evaluation of acceptability and applicability form. Figure S2: Customised GRADE Evidence to Decision template. [file 13047_2022_533_MOESM1_ESM.docx]
